# Black Silicon Surface-Enhanced Raman Spectroscopy Biosensors: Current Advances and Prospects

**DOI:** 10.3390/bios14100453

**Published:** 2024-09-24

**Authors:** Yaraslau Padrez, Lena Golubewa

**Affiliations:** Department of Molecular Compounds Physics, State Research Institute Center for Physical Sciences and Technology, LT-10257 Vilnius, Lithuania; yaraslau.padrez@ftmc.lt

**Keywords:** black silicon, surface-enhanced Raman spectroscopy, biosensors, sensitivity, enhancement factor, biocompatibility

## Abstract

Black silicon was discovered by accident and considered an undesirable by-product of the silicon industry. A highly modified surface, consisting of pyramids, needles, holes, pillars, etc., provides high light absorption from the UV to the NIR range and gives black silicon its color—matte black. Although black silicon has already attracted some interest as a promising material for sensitive sensors, the potential of this material has not yet been fully exploited. Over the past three decades, black silicon has been actively introduced as a substrate for surface-enhanced Raman spectroscopy (SERS)—a molecule-specific vibrational spectroscopy technique—and successful proof-of-concept experiments have been conducted. This review focuses on the current progress in black silicon SERS biosensor fabrication, the recent advances in the design of the surface morphology and an analysis of the relation of surface micro-structuring and SERS efficiency and sensitivity. Much attention is paid to problems of non-invasiveness of the technique and biocompatibility of black silicon, its advantages over other SERS biosensors, cost-effectiveness and reproducibility, as well as the expansion of black silicon applications. The question of existing limitations and ways to overcome them is also addressed.

## 1. Introduction

**Figure 1:**
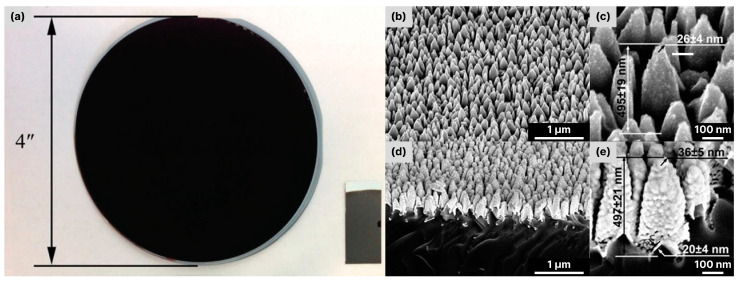
(**a**) Photo of a 4″ (100 mm) black silicon wafer and a sample of black silicon coated with Ag. Adapted with permission from Ref. [17] Copyright © 2024, John Wiley and Sons. (**b**–**e**) SEM images of black silicon (**b**,**c**) and black silicon coated with Au (**d**,**e**) [18]. Adapted with permission from Ref. [18] Copyright © 2024, American Chemical Society.

Silicon is undoubtedly one of the dominant semiconductor materials used in electronics and photonics. In contrast to polished silicon, which is highly reflective in the UV-to-NIR spectral range [1,2], silicon with a micro- and nanostructured surface is characterized by significantly altered optical properties. In particular, its reflectance decreases (and absorption increases) down to several percent [3] in the whole range from the UV to the NIR [4,5]. In some cases, the absorption of such micro-structured silicon reaches 99.5% in the spectral range of 350–2000 nm and about 99.8% in the spectral range of 1000–1250 nm [6], which gives this material a deep black color. Therefore, the highly absorbing micro-/nanostructured silicon was called “black silicon” (Figure 1a). The micro- and nanostructures of black silicon can have various shapes, including pyramids, needles, cylinders, nanowires (NWs), holes, pores, etc. [7] (Figure 1b,c). The main purpose of these structures is to efficiently trap light [8], i.e., to create conditions for numerous reflections between the sidewalls of the structures and transmission events at the boundaries of the microstructures, as a result of which the specular reflection of the parent material is almost completely suppressed. The structural defects in the silicon lattice or impurities (e.g., sulfur) that normally occur during the micro-structuring process also contribute to increased absorption [7,9], especially absorption in the NIR spectral range.

Black silicon was not originally produced for a specific purpose but was an unwanted waste product generated in the silicon industry [10]. However, its special optical properties have drawn the attention of researchers to this material. Black silicon’s wide-range absorbance, cost efficiency, ease of fabrication and ease of integration into existing electronic devices have made it a promising material for solar cells [9], visible light and NIR sensors [9,11], THz emission sources [12], microfluidic devices [13] and NIR photodetectors, where black silicon replaces indium gallium arsenide and germanium [14]. It has been shown that the additional treatment of black silicon, such as Se-hyperdoping or chemical functionalization, imparts sensory properties to this material and allows its use for the selective detection of ammonia [15] and nitroaromatic compounds with a detection limit of 10^−12^ ppt [16].

Currently, the application of black silicon is shifting from gas sensing or light detection to biosensing and the detection and characterization of biochemical compounds. In general, biosensors unite a large class of devices that are able to recognize and amplify the signal originating from a biological process/object and convert it into a detectable, often electrical or optical signal [19]. Many systems already exist, but each of them has limitations that could be obviously overcome if the system is changed for or accomplished with black silicon.

Optical detection methods are the most favorable and least invasive for bio-applications, as the light is collected from a certain distance. They are often based on the detection of fluorescence or scattering of the incident light by the biological object. However, the interaction of light and biological objects (biomacromolecules, living cells, tissues) is a complex process [20], and the meaningful signal is often weak and hidden in the autofluorescence background or in elastic scattering [21]. The probability of inelastic scattering, such as Raman scattering, is lower than that of elastic scattering by a factor of about 10^6^ and therefore hardly detectable [20]. Nevertheless, Raman spectra can provide much more meaningful information about the sample than, e.g., fluorescence, as Raman spectra consist of the bands attributed to the characteristic vibrations of the molecules and serve as a “fingerprint” of an analyte [22]. The typical Raman scattering cross section for biomacromolecules is about dσ/dΩ~10^−30^–10^−25^ cm^2^ sr^−1^ [23]), and an acceptable Raman signal can be obtained from only highly concentrated solutions, containing, for example, >10 mg/mL proteins [24]. The low probability of Raman scattering is one of the main drawbacks of the method. Despite this, the detection of Raman spectra of the analytes has become the key to the development of biosensors based on black silicon.

The sensitivity and efficiency of Raman measurements can be significantly improved by using special substrates consisting mainly of noble metal nanostructures (Ag, Au, Cu, Al [25]). Incident light induces collective dipolar oscillations (or surface plasmons, SPs) of the conduction electrons in metal nanostructures. When the frequency of the incident light and eigenfrequencies of the SPs coincide, surface plasmon resonance (SPR) occurs. If the size of the plasmonic nanostructures is smaller than the wavelength of the incident light, the SPR is called localized (LSPR). It leads to a significant increase in the local electromagnetic field near the surface of the metal nanoparticle and the creation of so-called “hot spots”. The latter support the amplification of the Raman signal of the molecules of the analyte adsorbed on or localized near the metal surface by a factor of 10^2^–10^14^ [26] and enables surface-enhanced Raman scattering spectroscopy (SERS) to detect extremely low concentrations of molecules with very high precision and accuracy.

One of the most important prerequisites for the successful application of the SERS method, which is also one of the weak points of SERS, is the development of a stable, scalable substrate with a controllable distribution of hot spots and predictable amplification to ensure reliable and reproducible results. The electrochemically roughened electrode surface, which was historically the first SERS substrate, is characterized by random patterning and uncontrollable amplification [27]. Colloidal solutions of various nanoparticles, including noble metal [28], magnetic [29], core-shell dielectric-metal [30], metal–metal [31] and metal–dielectric/oxide/polymer [32]), when used as synthesized or immobilized on a solid flat substrate [33,34,35,36,37], although offer the significant enhancement of Raman intensity, suffer from poor stability [38], dependence on storage conditions [39] and poor uniformity [40,41]. The direct production or in situ growth of regularly repeated noble metal nanostructures should solve the problem of repeatability but is not cost-effective. The direct fabrication of noble metal nanoparticles on the 3D support structures has proven to be a solution to this problem. Pillar arrays [42], grooves [43], wires [44], holes [45], etc., in Si, SiO_2_, glasses, etc., have significantly increased the effective surface area and favor the formation of multiple noble metal nanostructures by, e.g., magnetron sputtering [46], evaporation [47], etc.

In this context, the use of black silicon as a micro- and nanostructured 3D precursor substrate for the formation of plasmonic nanoparticles responsible for the amplification of Raman signals has attracted much attention in recent decades. The possibility of achieving semi-regular structures using various etching techniques without additional masking, the controllable surface geometry, the optical and electronic properties, the possibility of predicting the amplification of the Raman signal, the easy integration of black silicon into electronic devices, lab-on-chip systems, microfluidics, etc., make black silicon a suitable and cost-effective material for the development of SERS substrates and to fulfill the requirements of an SERS biosensor.

In this review, the prospects and advantages of using black silicon as a material for various biosensors are considered from different angles: fabrication, cost efficiency, sensitivity, SERS performance, an occupied niche and promising directions still open. The fabrication of black silicon is considered in terms of cost, simplicity, scalability and suitability for the development of a higher-performance SERS biosensor. The dependence of SERS efficiency on the surface morphology of the black silicon and the mechanisms of Raman signal enhancement, which are determined by the micro-structuring of the silicon surface and the quality and parameters of the noble metal coating, are discussed. The issues of the biocompatibility of black silicon and non-invasiveness of SERS measurements with black silicon are considered in the context of the advantages of this material over other SERS biosensors. Finally, the cost-effectiveness, long-term utilization, reproducibility of results and prospects for the transition of the black silicon-based SERS substrate from laboratory routine to real-world applications are discussed.

## 2. SERS Biosensor: Key Prerequisites for Best Performance

The development of materials for biosensor technology is subject to several strict requirements that must be met. These materials come into contact with a biological sample, e.g., living cells, tissues, biological fluids, such as blood and biomacromolecules (proteins, DNA, RNA, etc.), and must therefore ensure good biocompatibility, be inert to the biological sample and not cause chemical or physical damage [48], be sterile, be stable in different environments (buffer, serum, etc.) and preferably be integrated with silicon photonics. The proper surface stiffness, geometry, its micro- and nanostructuring [48,49,50,51,52] and surface chemistry [53] are crucial to minimize surface-related adverse effects and to ensure the integrity of the biological sample.

In SERS biosensors, the substrate represents the interface between the sensor and analyte and is the key component that determines the SERS performance. The SERS technique utilizes the local field enhancements that occur primarily on metallic plasmonic surfaces under the LSPR conditions to amplify the Raman signal from analyte molecules located at or near the surface [54,55]. Although SERS is also possible with non-plasmonic substrates [56], the SERS performance of such materials is significantly lower than that of plasmonic ones.

**Figure 2:**
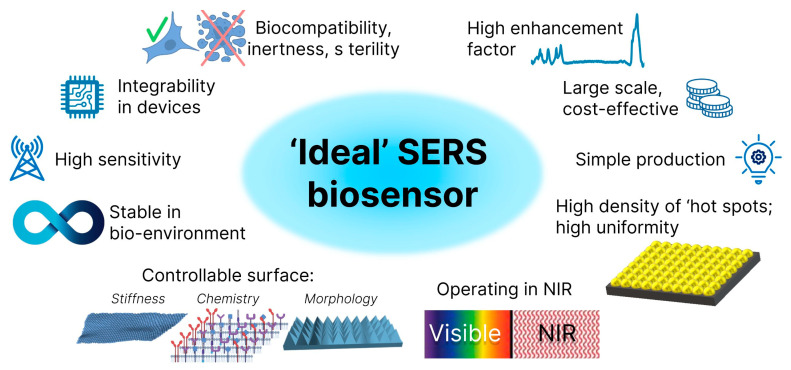
Requirements to an SERS biosensor.

In principle, only plasmonic nanostructures, such as Au, Ag, Cu, Al and a suitable excitation source with a wavelength that matches the LSPR of the particles, are required to enable SERS sensing. The apparent simplicity of an SERS sensing system has led to the emergence of various SERS substrates. However, all SERS systems have very different properties, and only some of them can be successfully used for biosensing. Some of the most important SERS properties and parameters, which in combination could describe a SERS substrate that comes close to an “ideal” biosensor, are shown schematically in Figure 2 and are briefly explained below:

- *SERS performance*. The main quantitative parameter that allows for a comparison of the efficiency of various substrates is the enhancement factor (EF). An experimental EF describes the dominance of the SERS signal over the Raman signal of analytes obtained under non-SERS conditions. The experimental estimation of the SERS substrate EF is often based on the following formula [57]:

EF=ISERSNSurfIRSNVol,

where I_SERS_ is the intensity of the SERS signal of the analyte molecules, N_Surf_ is the number of analyte molecules adsorbed on the surface, N_Vol_ is the average number of analyte molecules in the scattering volume for the non-SERS measurements and I_RS_ is the Raman signal intensity for the measurements under non-SERS conditions [58]. This calculation is convenient to compare SERS enhancement across different substrates, but the accuracy is limited as it is often difficult to estimate the exact number of molecules adsorbed on the surface. The EF also depends on the excitation wavelength and the band in the spectrum of the analyte for which it is calculated, which makes it difficult to compare results from different studies. There are also several modifications of EF, such as the single-molecule enhancement factor (SMEF) or the analytical enhancement factor (AEF), which are less convenient for substrate comparisons [59]. Furthermore, the comparison of the SERS EF of static substrates, such as immobilized plasmonic nanoparticles and dynamic substrates (colloids), is not correct [60].

- *High density of “hot spots”*. The SERS signal is generated by the molecules of the analyte in the areas of very large local field enhancements, the “hot spots”. The more molecules are “trapped” with hot spots and excited with the laser, the better the sensitivity [61,62].
- *High uniformity and reproducibility of SERS EF*. Although high uniformity of SERS EF is not required for some studies, such as single molecule detection, it is needed for quantitative analysis. For such analysis, for example, nanostructured silicon covered with silver nanostructures is a perfect candidate as it allows for SERS with a low relative standard deviation over large areas and provides the possibility of balancing the hot spots with extreme EF by normalizing the signal to the Raman intensity of the silicon band at 520 cm^−1^ [63].
- *High sensitivity (limit of detection, LOD)*. Nano-to-femtomolar concentrations of bio-analytes circulating in blood and serving as biomarkers of various diseases dictate the corresponding requirements to SERS biosensors.
- *High stability.* As SERS is a very sensitive technique, SERS substrates and SERS-active materials, such as colloids of nanoparticles, often suffer from poor stability [38] and strong dependance on the storage conditions [39] and quickly degrade and lose their SERS properties.
- *Simplicity* in fabrication, large scale, cost-efficiency. Most SERS substrates and materials are disposable. Therefore, simplicity, low cost and large-scale production are extremely important parameters that would facilitate the transition of SERS sensing from the laboratory to practice.
- *Operation in NIR*. NIR irradiation is more favorable for the analysis of biological objects, especially cells and tissues as, in contrast to UV and even visible light, it does not cause sample damage.

The variety of fabrication approaches, ranging from complex processes to simple one-step methods, the different designs of plasmonic nanostructures deposited on black silicon, the controllable 3D geometry, light trapping and other optical properties, as well as the chemical and biological inertness of pure silicon, make it possible to demonstrate the potential of black silicon as one of the best candidates for combining all the properties of an “ideal” SERS biosensor in one substrate. Further sections discuss, generalize and summarize the existing data on black silicon fabrication, SERS performance and current applications for the SERS sensing of various biological samples.

## 3. Black Silicon Fabrication and Metal Deposition: Meeting the Requirements for SERS

The micro- and nano-structuring of silicon significantly changes the absorption of the material [3], and the specular reflection of light can be reduced by more than 90% in the visible and NIR range [6]. Nano/micro-structures with different geometries create one of the key conditions for the increased absorption of light. Multiple reflections at the interfaces of the neighboring micro- and/or nanostructures are accompanied by corresponding transmissions, which lead to a subsequent reduction in the intensity of the incident light [64]. Such an effect is known as light trapping [65,66]. Additional silicon doping, performed either independently or simultaneously during the micro-structuring process [7,9], can also lead to an increase in absorption by black silicon and is explained below.

It is important to note that micro- or nanostructured silicon with high absorption in a broad spectral range is not always referred to as black silicon. When it comes to ordered arrays of identical structures, often produced by complex methods, authors often do not use the term “black silicon” but terms that emphasize the orderliness of the structures and their shape (e.g., an array of cones [67], micro-pillars [68], an array of nanoneedles [69], etc.). However, we will consider these structures together with those referred to as “black silicon”, as these silicon structures have high absorption of the same origin as black silicon.

The geometry and properties of the created structures are determined by the method by which they were produced, which in turn affects the efficiency of the black silicon when used in SERS. The most widely used top-down methods of nano- and micro-structuring of silicon are electrochemical etching [70,71,72,73], metal-assisted chemical etching (MACE) [74,75,76,77] and reactive ion etching (RIE) [78,79,80,81], etc., considered as the most convenient and low-cost, femtosecond/nano-second laser-assisted fabrication [8,82], laser chemical etching [7] and plasma-less atmospheric dry etching [83]. The latter has only recently been introduced but has already been proven to be a cost-effective technique to produce nanoscale silicon surfaces. These methods enable uniform coverage of the surface with nano- and microstructures on a large scale, and the creation of a highly developed surface is mainly achieved by a chemical reaction with silicon, which causes selective erosion of the silicon wafer surface [84]. The detailed description of various production methods of black silicon can be found, for example, in reviews [10,85,86,87,88]. In this review, we focus on the methods used to produce black silicon, with an emphasis on the formation of properties in black silicon that are suitable or necessary for SERS.

### 3.1. Electrochemical Etching (Or Electrochemical Anodization)

**Figure 3:**
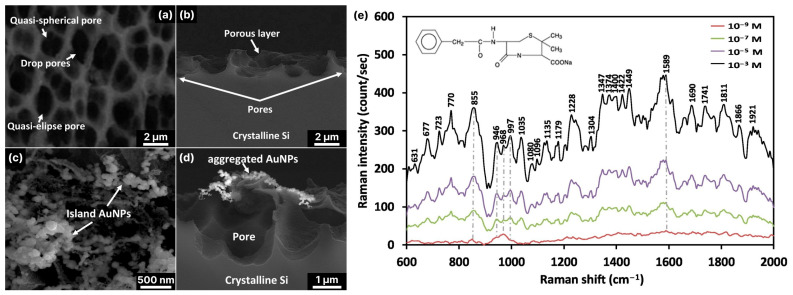
(**a**) FE-SEM of the PSi surface morphology, (**b**) cross-sectional FE-SEM image of PSi substrate, (**c**) FE-SEM image of the Au NPs deposited on the PSi, (**d**) cross-sectional FE-SEM image of Au NPs/PSi, (**e**) SERS spectra of PG antibiotic at different concentrations adsorbed on a Au NPs/PSi SERS active substrate [72]. Adopted with permission from Ref. [72] Copyright © 2024, Elsevier B.V.

Electrochemical etching (or electrochemical anodization) mainly results in porous, black silicon [86], and the fabrication of 3D micro- and nanostructures often requires an additional photolithographically defined mask [89], but fabrication without a mask is also possible [71]. Etching is often carried out in a mixture of HF, H_2_O and ethanol (or DMSO [73]), with a silicon wafer attached to the anode [90]. The resulting structures are arrays of pore channels of different sizes and geometries (for an example of electrochemically etched silicon see Figure 3a). The pore size and depth are controlled by the current density, concentration of HF, etching time and illumination when the n-doped Si wafer is used [72]. The latter is probably the reason for the choice of mainly p-type silicon to produce black silicon, as it does not require additional illumination. Some representative sets of fabrication conditions used in SERS studies by different authors are listed in Table 1.

To endow the porous silicon with SERS properties, the formation of plasmonic nanostructures is required. Covering the porous silicon with a continuous metal layer serves to create the “hot spots”, which are metal voids but not nanoparticles in the case of porous materials. D. Sigle et al. [91] showed that for 100 nm aluminum voids, the enhancement of the local electric field occurs only in the edge (rim) region, while the increase in the void (pore) size leads to the appearance of additional plasmonic modes inside the void. The latter means that the Raman signal of the analyte trapped in the pore is amplified. The porous silicon is often covered with metals either via electrochemical coating or through the electroless immersion of the porous silicon in salt solutions or via a combination of both. For example, K. Artsemyeva et al. [71] performed hybrid metallization of porous silicon by depositing Ni electrochemically in an aqueous solution of NiSO_4_/NiCl_2_/H_3_BO_3_/saccharin at a current density of 10 mA/cm^2^ for 7 min and then electrolessly depositing Ag by immersing the substrate in an aqueous solution of AgNO_3_/HF (45%)/C_2_H_5_OH. They have shown that such hybrid metallization results in better SERS enhancement than Ag coating alone on the porous silicon.

**Table 1:** Comparison of the parameters and SERS efficiency of the black silicon-based SERS substrates produced via electrochemical anodization and metal-assisted chemical etching (MACE).

Black Silicon Fabrication	Si Wafer Parameters	Black Silicon Porosity, Morphology	Metal, Thickness, Layer Type	Metal Deposition Method	Enhancement Factor	λ_ex_, nm	Detected Object, Detection Limit	Reference
**Electrochemical anodization**								
HF (45%): C_2_H_5_OH (1:1), 10–50 mA/cm^2^, 25 min. H-termination: in HF (3.3%), 5 mA/cm^2^, 40 s	(100) boron doped, 0.02 Ω × cm	Porous silicon: pore diam. 20–40 nm, depth 1.5 μm	– – –	No metallization	N/A	532	R6G, 3.2 × 10^−8^ M	[70]
HF (45%): DMSO (10:46), 8 mA/cm^2^, 7 min	(100) boron doped, 12 Ω × cm, size 100 mm	Macro-porous silicon (spongy structure): pore diam. 1 μm, depth 2.5–2.7 μm	Ag; Electrodep. Intermediate Ni; Ag NPs 10–150 nm	Ag (electroless) MID; 5–120 min	10^4^ (Ag); 10^5^ (Ni/ Ag)	441.6 514.5	CuTMpyP4; R6G, 10^−11^ M	[71]
HF:C_2_H_5_OH (1:1), 20 mA/cm^2^, 8 min, irradiation: 630 nm, 30 mW/cm^2^	(100) n-type, 10 Ω × cm; 1.5 × 1.5 cm^2^	Macro-porous: pore diam. 0.75–3.25 μm, depth 2.5 μm, porosity 55%	Au NPs, 50 nm, aggregated	Au MID: HAuCl_4_ (1 mM): HF (3 M)	5 × 10^7^	532	Penicillin G, 10^−9^ M	[72]
HF (45%): H_2_O:C_3_H_7_OH (or DMSO) (1:3:1), 7–80 mA/cm^2^	Monocrystalline Si, 3 × 3 cm^2^	Mesoporous: depth 1 μm, porosity 80–85% Macroporous: depth 3 μm, porosity 60–65%	Cu NPs, on top of the pores, 20–280 nm	Cu DID: CuSO_4_ × 5H_2_O + 5 mM HF or CuSO_4_ (25 mM): HF (5 mM): C_3_H_7_OH (0.1 M)	N/A	441.6 532	CuTMpyP4, 10^−6^ M	[73]
HF (18.7 M); (i) Pentanol: butanol: ethane = 1:0.25:0.25 (ii) *n*-Propanol (iii) Isobutanol (iv) Acetonitrile (v) Ethanol (vi) tert-Butanol (vii) *n*-Butanol	(100) p++ type, boron doped	Porous	Au, rough layer: (i)-(vi) 30 nm (vii) 10 nm, 30 nm, 50 nm, 100 nm, 200 nm, 300 nm	Au PVD	10^8^ (p-MBA)	532, 785	*p*-MBA (10^−6^ M), human blood, cerebrospinal fluids, urine	[92]
HF: water: ethanol 25:25:50 10 mA/cm^2^, 5 min	(100) boron doped 5–10 Ω × cm	Meso-porous silicon, pore diam. 10–20 nm	Incorporate Au NPs, 20 nm	Au (electroless) MID: HF (C = 0.15 M): Gold (III): Chloride (AuCl_3_) (C = 1 mM), 3 min	N/A	633	MCF7 breast cancer cells	[93]
**Metal-assisted chemical etching (MACE)**								
Two-step MACE Au pre-coated (3 nm thick). Etching: HF:H_2_O_2_:C_2_H_5_OH (1:1:1), 5, 10, 15 min	(100) p-type; 1–10 Ω × cm, 6‘‘	Porous silicon: pore diam. 0.52–0.76 μm, depth 2.53–5.39 μm, porosity approx. 41–45%	Decoration with Ag NPs	Ag NPs MID	6 × 10^7^ (R6G)	532 633	Melamine, 10^−9^–10^−5^ M; R6G, 10^−9^–10^−5^ M	[74]
Two-step MACE Ag pre-coated: HF (4.6 M): AgNO_3_ (0.44 M), 10 s. Etching: HF (4.6 M): H_2_O_2_ (0.44 M)	(100) boron-doped, 1–10 Ω × cm, 1 × 1 cm^2^	SiNW arrays: depth 150–300 nm	Au NPs 10–20 nm. Au backplane 10 nm.	Prior OAD-Ag removal: HNO_3_; OAD NPs, Au metal backplane	1.8 × 10^6^	-	MG, 10 nM	[75]
Two-step MACE Ag pre-coated for 0.5, 1, 3, 10 min. Etching: water-based HF (4.36 M): H_2_O_2_ (0.23 M), 60 min.	(100) p-doped (n-type), b-doped (p-type), 4’’	SiNWs arrays: length 2–10 μm (p-SiNWs), length <4 μm (n-SiNWs)	Ag, Au; NPs 75 nm, Ag NPs between SiNWs, Ag/Au dendrites 300–500 nm	(i) No metallization; (ii) Electroless Au MID: HAuCl_4_ (3 mM): HF (0.15 M): C_2_H_5_OH (1.5 mM)	N/A	633	R6G, DTNB; 10^−9^–10^−6^ M	[76]
Two-step MACE Ag NP pre-coated: HF (5.55 M): AgNO_3_ (0.015 M), 5 s. Etching: 5 mL HF (48%): 2 mL H_2_O_2_ (50%): 23 mL deionized H_2_O, 10 min.	(100) p-type, n-type, 0.01 Ω × cm	Mesoporous SiNWs	Ag NPs 38.9 nm, between/on SiNWs	Prior Ag NP MID-residual Ag removal: HNO_3_ (10%) Electroless Ag MID: AgNO_3_ (15 mM): HF (5.55 M), a few s	10^8^ (MB); 10^9^ (RB)	514	MB, RB; 10^−12^ M	[77]
Two-step MACE Au NPs pre-coated. Etching: H_2_SO_4_ (8%): H_2_O_2_ (37%) (3:1), 10 min	Crystalline Si	SiNWs: Pore diam. 100 nm; depth 34–35 µm porosity 55–83%	Au NPs 10 nm, between/on SiNWs	Prior Au MID-residual Au removal: HCl: HNO_3_ (3:1). Electroless Au MID: HAuCl_4_/HF, 10, 20, 30 s.	6.1 × 10^4^	633	MB, 10^−15^ M	[94]
Single-step MACE Etching: aqueous solution of AgNO_3_:HF, 2, 3.5, 5, 30 min.	(100) p-type	Si NWs: depth 200–300 nm (2–5 min MACE), depth 6 μm (30 min MACE)	Ag NP aggregates, at the SiNWs tips, a few Ag NPs between/on SiNWs.	(i) No metallization (2, 3.5, 5 min MACE); (ii) Prior MID – residual Ag removal: HNO_3_, 4 min (5- and 30-min MACE); Electroless Ag MID in MACE etching solution, 3–10 s;	10^5^–10^10^	514	R6G 10^−13^ M	[95]

Abbreviations—R6G (rhodamine 6G), MB (methylene blue), RB (rose bengal), MG (malachite green), CuTMpyP4 (Cu(II)-tetrakis(4-N-methylpyridyl) porphyrin), DTNB (5,5′-dithiobis (2-nitrobenzoic acid)), NP (nanoparticle), SiNW (silicon nanowire), MID (metal immersion deposition), DID (displacement immersion deposition), OAD (oblique angle deposition), PVD (Physical Vapor Deposition), *p*-MBA *(p*-mercaptobenzoic acid), MCF7 (human breast cancer cell, Michigan Cancer Foundation).

Although Ag metallization is the most widely used technique as Ag defines superior SERS properties, copper is also used to create plasmonic nanostructures. Cu immersion deposition is performed in the presence of HF, which removes SiO_2_, promotes the substitution of Si atoms by Cu and leads to the formation of a copper film with copper nanoparticles of a controllable size [96] both on the surface of porous silicon and inside the pores. In [73], using 10^−6^ M Cu(II)-tetrakis(4-N- methylpyridyl)porphyrin (CuTMpyP4) as a test solution, it was shown that Cu nanostructures prepared through Cu displacement deposition on porous silicon enhance the Raman signal, while similar Cu nanostructures without porous silicon support are SERS-inactive. This fact indicates the important contribution of the silicon substrate to SERS, although an explanation was not given.

Although the process of electrochemical etching of silicon with subsequent metallization through immersion deposition seems simple and easy to implement, there are several technological obstacles that limit its use for SERS biosensing. The porous silicon is highly hydrophobic with a contact angle of about 150° for water [97]. The hydrophobicity is caused both by the porosity of the material (Lotus effect [98]) and by the surface modification during etching. The latter is caused by the reaction of the Si surface with HF and C_2_H_5_OH, which are present in the etching medium, and the formation of mostly non-polar Si–O–Si, –OSiCH_3_ and Si–F/Si=F_2_ groups on the surface [73]. The high hydrophobicity prevents aqueous salt solutions from wetting the surface well and penetrating into the pores, which means that the porous silicon is not covered with metal and no hot spots are created. This significantly reduces the success rate in the production of SERS substrates with a high-quality, uniform distribution of hot spots and controlled amplification of the Raman signal. Metallization through immersion deposition often leads to the agglomeration of the nanoparticles (see Figure 3a–d). The gaps between the nanoparticles in these agglomerates serve as “hot spots” and can also enable a significant amplification of the Raman signal. For example, porous silicon prepared via the photoelectrochemical etching of n-type silicon followed by gold immersion deposition was shown to have an enhancement factor of >10^7^ and a detection limit of penicillin G in the nanomolar range (Figure 3e) [72].

### 3.2. Metal-Assisted Chemical Etching (MACE)

The technique of metal-assisted chemical etching (MACE) is widely used because it enables the production of black silicon on a large scale [99]. In the MACE process, silicon is etched from the surface of a Si wafer in the presence of HF and an oxidizing agent (e.g., H_2_O_2_), with the oxidation reaction catalyzed by noble metals, such as gold, silver, aluminum, copper and nickel [100]. The latter fact can significantly contribute to SERS applications and will be additionally considered below. In general, MACE can be carried out as a one- or two-step process.

**Figure 4:**
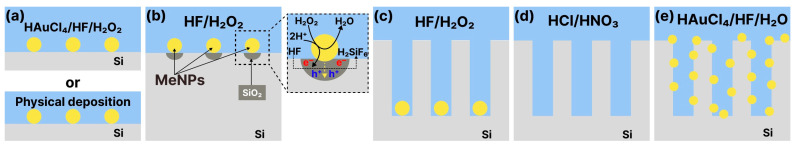
Sequence of the two-step MACE process: (**a**) deposition of noble metals either via immersion deposition or by physical deposition (magnetic sputtering, evaporation, etc.), (**b**) initial phase of etching in HF/H_2_O_2_ aqueous solution, (**c**) end of etching followed by (**d**) removal of metal catalysts with acids, (**e**) metal immersion deposition of NPs for further SERS applications. Modified from [94].

In a single-step MACE, the metal ions that serve as catalysts are already present in the etching solution. Silver nitrate AgNO_3_ or tetrachloroauric(III) acid HAuCl_4_ is added to the mixture of HF and H_2_O_2_ [95]. The dissociated metal ions serve as acceptors for electrons from the valence band of the silicon and, after their reduction, form nucleation sites of nanoparticles on the surface of the silicon. Simultaneously with the reduction of the metal ions, under the sites of nanoparticle nucleation, the formation of holes in the silicon leads to its oxidation. Hydrofluoric acid HF removes the silicon oxide, and the metal catalyst migrates deeper into the resulting pore. The process continues iteratively, and the depth of the pore increases and is limited by the etching time and depends on the etchant composition and etching temperature [101]. The noble metal nanoparticle finally ends up at the bottom of the pore and can be removed by additional treatment with, e.g., HNO_3_ [102]. However, the latter is not necessary and is perhaps even superfluous in SERS applications of MACE-produced black silicon, since the fabricated black silicon requires additional metallization. The nanoparticles in the pores, which are obtained as a by-product of MACE, can already serve as hotspots for SERS [76,95]. The main difference between the two-step MACE process and the one-step process is that in two-step MACE, the silicon surface is first coated with noble metal nanoparticles by any physical (e.g., magnetron sputtering, electron beam evaporation) or more often chemical process, such as electroless metal deposition [74,76]. This step aims to form noble metal nanoparticles or nano-islands on the silicon surface, which later will serve for the chemical etching of the silicon surface. The etching process is similar to the one-step MACE process [101], with the exception that no silver- or gold-containing compounds are present in the etching solution. The principal sequence of the two-step MACE is shown in Figure 4.

**Figure 5:**
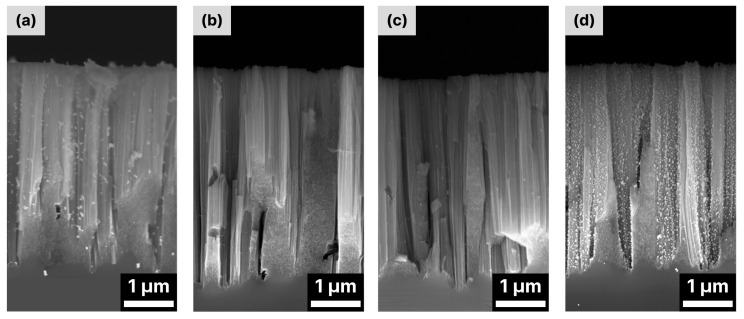
Cross-section scanning electron microscopies of bare (**a**) and surface-modified (**b**–**d**) Si NWs decorated with Au NPs. Surface modifications: (3-aminopropyl)triethoxysilane (**b**), (3-mercaptopropyl)trimethoxysilane (**c**), and (3-glycidiloxypropyl)trimethoxysilane (**d**). Adapted from [104].

The two-stage MACE process is a more controllable process than electrochemical etching and enables the production of more diverse micro- and nanostructures, including silicon nanowires [94] (Figure 5a), silicon pores [74,77], nanorods, nanocones, nanopillars [103], etc. Since the erosion of the silicon takes place mainly under the nanoparticles, the diameter, shape of the pores and porosity are completely determined by the shape, geometry and spacing between the noble metal nanoparticles deposited on the silicon surface prior to etching. An intermediate step in which the silicon is treated with HNO_3_ between metal deposition and etching can be added. It allows for an increase in the distance between the deposited metal nanoparticles by “washing” the excess nanoparticles with acid. This makes it possible to obtain silicon with a lower porosity [102]. As a result, the obtained types of black silicon vary from porous with spatially separated voids to densely packed silicon nanowires (SiNWs) when the initial density of the deposited metal nanoparticles is high. In addition, the shape of the apexes of the silicon structures can be sharpened to obtain cones or a pencil-like shape by repeatedly exposing the nanopillars obtained through two-stage MACE to a mixture of AgNO_3_, HF and HNO_3_ or H_2_O_2_, resulting in selective deposition of Ag nanoclusters on the tips and their subsequent removal, which can be figuratively compared to sharpening a pencil [103].

The possibility of avoiding additional metallization of the black silicon produced with MACE, as the noble metal nanoparticles between the nano/microstructures are naturally formed during the MACE process, makes this technique very promising, cost-effective and easy to handle. In [76], it was shown that SiNWs obtained via MACE exhibited satisfactory enhancement of the Raman signal of rhodamine 6G (R6G) in a solution with a concentration of 10^−6^ M. The additional electroless deposition of Au dendrite-like nanostructures improved the enhancement and allowed the detection limit of 10^−9^ M to be reached. Similar results were obtained by I. Kachylas et al. [95], where the authors were able to detect R6G molecules from 10^−8^ M solutions with silicon nanowires prepared via a one-step MACE without additional metallization. However, they also report that purification of the SiNWs with HNO_3_ from residual Ag nanoparticles and the subsequent additional deposition of Ag nanoparticles leads to an improvement of the detection limit to 10^−13^–10^−10^ and to enhancement factors in the range of 10^5^–10^10^. To improve the decoration of SiNWs with metal nanoparticles, the additional functionalization of SiNWs can be performed. In [104], the SiNWs produced with MACE were first washed with an Au etching solution to remove the Au residues remaining from the production process. They were then coated with different polymers and decorated with Au NPs by immersing the bare and polymer-coated SiNWs in an Au colloid solution. As a result, the surface modification with (3-glycidiloxypropyl)trimethoxysilane significantly improved the decoration of the polymer-modified SiNWs with Au NPs (Figure 5d) compared to the bare SiNWs (Figure 5a), while other polymers had the opposite effect and prevented the decoration of the SiNWs with Au NPs (Figure 5b,c).

The black silicon produced with MACE is also characterized by a high hydrophobicity, which has the same origin as that of the black silicon produced via electrochemical etching [74]. This property can restrict the access of the analyte molecules to the noble metal nanoparticles located at the bottom of the pores. Therefore, additional metal deposition is often required, using methods similar to those used after electrochemical etching, e.g., electroless immersion deposition with AgNO_3_ or HAuCl_4_ solutions. For example, [105] micro-/nano-nested structures of silicon obtained via MACE were additionally covered with gold nanoparticles and then were installed into a microfluidic cell demonstrating a satisfactory enhancement factor of around 10^6^ and staying in good condition for almost 120 days of storage. C. Tsao et al. [74] obtained highly hydrophobic porous black silicon by etching silicon wafers precoated with Au in an HF/H_2_O_2_/C_2_H_5_OH aqueous solution and additionally decorated it with Ag nanoparticles via electroless Ag deposition, which imparted hydrophilicity to the black silicon and defined the enhancement factor of 10^6^ estimated for R6G. R. Ghosh et al. [77] were able to achieve an even higher enhancement factor of 10^8^–10^9^ using the two-step MACE method for the production of mesoporous silicon nanowires decorated with Ag nanoparticles, with the detection limit of the substrate being around 10^−12^ M.

Despite the hydrophobicity of black silicon produced with MACE, the high fragility of thin and long structures, such as nanowires, is also one of the disadvantages of black silicon of this type. Long silicon nanowires tend to conglomerate due to the presence of dangling bonds and electrostatic charges characteristic of high-aspect-ratio nanostructures [76]. This can significantly limit the amplification of the Raman signal by blocking the molecules’ access to the metal nanoparticles between the nanowires and reducing the lifetime of the black silicon SERS substrate. For example, SiNWs fabricated through the combination of nano sphere lithography and MACE followed by gold deposition demonstrated significant Raman signal enhancement and the detection limit of about 10^−12^ M but suffered from the same problem of fragility [106].

Porosity influences the amplification of the Raman signal. In [94], the authors showed that reducing the porosity of silicon nanowires prepared with MACE from 83% to 55% leads to an increase in the intensity of the Raman signal by methylene blue molecules with a detection limit of 10^−15^ M, although the estimated enhancement factor is only about 6.1 × 10^4^. However, such a low value of the enhancement factor could be because the authors used the values of the molecule concentrations and not the number of molecules adsorbed on the nanoparticles and excited with a laser when calculating the enhancement factor. Estimating this number of molecules, in turn, is difficult to realize experimentally.

### 3.3. Inductively Coupled Plasma Reactive Ion Etching (ICP-RIE)

Inductively Coupled Plasma Reactive Ion Etching (ICP-RIE) is a dry anisotropic etching method for large-scale black silicon production that is actively used to produce diversified black silicon for SERS. In ICP-RIE, a plasma of highly reactive ion species (often SF_6_/O_2_ plasma) reacts with the surface of the silicon wafer and causes its selective erosion [84]. There are several modifications of the ICP-RIE method: “mixed mode etching” cryogenic ICP-RIE (simultaneous etching and passivation), room temperature ICP-RIE, the Bosch method (also known as DEM–“Deposit and Etch Many times”), DREM (“Deposit, Remove, and Etch Many times”) [107], etc. In cryogenic ICP-RIE, the cooling of the substate to temperatures below −80 °C is crucial for successful etching [107]. Black silicon structures can be produced without a mask (not any of the lithography steps) in the SF_6_/O_2_ plasma under over-passivating regime conditions, which means a high oxygen content compared to SF_6_ [108].

#### 3.3.1. Mixed-Mode Cryogenic ICP-RIE

**Figure 6:**
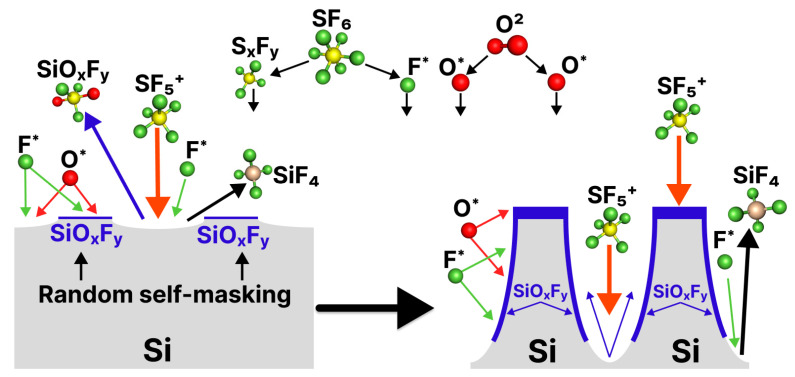
Schematic of the physicochemical mechanisms of anisotropic Si etching. S_x_F_y_*, F* and O* formed in the generated plasma are diffused to the Si surface. The passivation-layer (SiO_x_F_y_, blue color) random formation, the removal of bottom passivation layer and Si etching by F* occur simultaneously. The anisotropic behavior of the process enables the self-mask materials to define the shape and density of silicon structures. Modified from [112].

In cryogenic ICP-RIE, SF_6_ and O_2_ gases are used for the generation of F^•^ and O^•^ radicals, respectively [78]. The silicon substrate is often cooled down to about −110 °C. The reaction of F^•^ with the silicon surface results in the formation of SiF_x_ etching products (mainly SiF_4_) at the silicon surface, which further react with O^•^ radicals to form the passivation layer SiO_x_F_y_ on the cooled sidewalls of the silicon structures [109]. The passivation layer is then partially removed through the ion bombardment, and silicon becomes reopened for etching. The formation and destruction of the passivation layer of SiO_x_F_y_ determines the etching process. Since the ion bombardment is less intense on the side walls, the removal of the SiO_x_F_y_ layer is less effective there than in the bottom areas, which means that the etching mainly goes into the depths (see in Figure 6). One of the main advantages of the cryogenic ICP-RIE method is that the passivation layer is desorbed with increasing temperature, so that at the end of etching no impurities remain on the side surfaces of the microstructures, only pure silicon [109]. This is extremely important for SERS applications of black silicon. Minor impurities adsorbed on the surface of black silicon can generate a strong Raman signal that is amplified along with the signal of the molecule under investigation, making analysis much more difficult. Cryogenic ICP-RIE also enables the precise control of the surface geometry of black silicon nano/microstructures and their density by varying the gas composition and flow rates, temperature of the substrate, bias and RF power [17,110,111]. Further details about the deep ICP-RIE can be found in a review [112].

#### 3.3.2. Mixed-Mode Non-Cryogenic ICP-RIE

Recently, the large-scale production of grass-like black silicon was demonstrated by ICP-RIE in a mixture of SF_6_/O_2_ in the temperature range from −20 °C to +20 °C [113]. The samples of black silicon with vertical grass-like structures of up to 7 μm height and low reflection coefficients (3.1% at +20 °C and 1.28% at −20 °C sample temperature) were produced within only 5 min of etching. In contrast to cryogenic ICP-RIE, etching at room temperature (RT) and even at −20 °C is more desirable because operating at RT greatly simplifies the black silicon manufacturing process and reduces its cost. RT-ICP-RIE avoids the use of liquid nitrogen and significantly shortens the duration of the entire process, as operation at RT eliminates the time required for temperature stabilization of the sample.

#### 3.3.3. Mixed-Mode Room-Temperature ICP-RIE

J. Pezoldt et al. proposed the ICP-RIE method for the production of black silicon of the needle geometries at a substrate temperature of 20–30 °C [114]. In RT ICP-RIE, the mechanism of formation of the black silicon structures remains similar to that of cryogenic ICP-RIE. In short, under over-passivating conditions, SiO_x_F_y_ deposition occurs. In combination with poorly etched native silicon oxide, this leads to the formation of random masking spots on the bulk silicon (see Figure 6). Unmasked areas are etched through the reaction with F radicals and ion bombardment, while the sidewalls of the structures are self-masked with SiO_x_F_y_ and protected from etching. The result is needle-shaped (cone-shaped) structures with a height of 100–1000 nm, which are produced in 300 s [114].

#### 3.3.4. Bosch Method

The Bosch method (or DEM) [115] is a two-step process consisting of several successive cycles of isotropic etching with SF_6_ and the deposition of a fluorocarbon-based protective film, which is carried out by rapidly alternating between the input gasses. In order to obtain fewer rough sidewalls, the gas change should be fast. In addition, many etch/inhibition cycles must be performed to achieve an acceptable length of the structures. In contrast to continuous ICP-RIE, where etching/passivation provides a more conical shape of the structures, structures with more vertical walls can be obtained with the Bosch method [116]. The Bosch method and its modifications enable the production of cylinder-like structures with a controllable geometry. However, these structures are coated with a fluorocarbon film that is difficult to remove. This polymer film can significantly limit the SERS application of black silicon.

#### 3.3.5. SERS Performance of ICP-RIE Produced Black Silicon

Since continuous ICP-RIE (cryogenic or RT) allows one to obtain random, but quite frequent and densely arranged, conical structures, characterized by the absence of impurities, lower hydrophobicity and better accessibility for analyte molecules, complex cyclic methods, such as the Bosch method and its analogs, are not required for the preparation of SERS substrates, although SERS substrates with pillar-like SERS substrates were developed [116]. The RT ICP-RIE method, on the other hand, is superior to cryogenic modification because it is easy to implement and time- and cost-efficient [117]. In this respect, it is this modification of the method that has found wide application in the development of supporting 3D structures for SERS substrates (see Table 2).

In contrast to wet etching, where metallization is mainly performed through metal immersion deposition, in the case of ICP-RIE black silicon fabrication, the deposition of plasmonic noble metal nanostructures is mainly performed via (i) electron beam evaporation [17,79,81,118,119,124], resulting in either a non-continuous layer or spatially separated nanoparticles, (ii) the spin-coating of NPs [125], (iii) thermal evaporation [120], resulting in isolated metal islands, and (iv) magnetron sputtering [18,69,80,116,121], resulting in either a continuous rough metal layer or a mosaic-like pseudolayer. In many cases, however, the thickness of the metal layer deposited on black silicon exceeds 200 nm [2,69,79], which drives up the cost of producing the SERS substrate, although there has been a recent shift towards covering it with non-continuous pseudo-layers of noble metals of low thickness (in the tenths of a nanometer range) [18,116,124]. The reduction in noble metal thickness has no significant effect on the enhancement factor or detection limit of black silicon SERS substrates and is 10^6^–10^8^ [18,121] and 10^−9^ M [121], respectively. The latter is also achieved based on the diversity of silicon structures, which provide 3D support for plasmonic noble metal structures and allow for better access to hot spots than porous silicon. For example, arrays of silicon nanopillars fabricated with ICP-RIE and covered with noble metals enabled detection with an enhancement factor of about 10^11^ [68]. However, the substrate itself proved to be fragile, as the surface tension generated on contact with the analyte caused the nanopillars to break.

It should be mentioned that the production of silicon structures that are close in geometry to black silicon is also possible without ICP. In [123], a self-organized monolayer of polystyrene (PS) beads with a diameter in the sub-micrometer range formed on the silicon wafer served as the masking. The subsequent RIE with CF_4_:Ar (5:1) enabled the erosion of the silicon between the PS beads. However, degradation of the mask and thus contamination of the silicon structure may occur. In addition, metal deposition is complicated by the presence of PS beads at the top of the silicon structures, which must be removed before SERS measurements, and the risk of surface contamination remains. Although the structures fabricated with RIE also show affordable SERS performance, the complexity of fabrication and the high risk of surface contamination reduce their applicability for biosensing, leaving this scene for ISP RIE.

The high effective surface area, the various geometries, the cost-effectiveness of the ICP-RIE method, the absence of impurities, the simplicity of fabrication and the low consumption of noble metals have sparked interest in ICP-RIE and especially in RT ICP-RIE, as it offers stable, efficient and scalable substrates. One can speculate that the use of other dielectric materials, such as glasses, is cheaper, but the 40-fold higher etch rate of silicon via ICP-RIE [126] and the absence of charging effects that occur when etching dielectrics [127], which significantly impair directional etching and make 3D/2D structuring more difficult, removes all doubt.

Since electrochemical etching, MACE and various modifications of ICP-RIE are the most suitable methods to produce black silicon for SERS applications, we limit ourselves to these methods, although there are several attempts to produce black silicon on a large scale using other techniques, e.g., femtosecond laser ablation [128].

### 3.4. Selection of the Type of Silicon Wafer for the Production of SERS Substrates

Regarding the type of silicon most commonly used for production, i.e., n-type or p-type, the orientation of the silicon wafer ((100), (110) or (111)) and the resistivity, there is no uniform approach to selection. Both n- ([116,124]) and p-types ([71,74,120]) of crystalline silicon are used. However, it was shown in [76] that with similar fabrication, the reflectance of p-type black silicon is lower than that of n-type black silicon and that the length of p-type silicon nanostructures is longer than that of n-type silicon. Such a reduction in reflectance and the greater length of the silicon structures likely indicate more efficient light trapping, greater surface area and coverage with molecules of the analyte, resulting in greater SERS enhancement.

The silicon wafers with the crystal orientation (100) are mainly used for the production of black silicon (see Table 1 and Table 2). The orientation of the silicon wafer can also influence the etching performance and etching rate [99]. In contrast to plasma-assisted etching (ICP-RIE), where the vertical direction of etching is mainly determined by the silicon erosion by high-energy ions, plasma-free wet etching (such as MACE) leads to the formation of nano- and microstructures due to the different etching rates of the different crystal planes (orientation-dependent etching) [129]: the etching rate in the (111) direction is slower than that in (100) and (110), which is often explained by the different strength of the Si-Si bond in the different crystal planes. By varying the etching parameters, such as the ratio of [HF]/[oxidizing agent] concentrations, it is possible to obtain structures with different orientations [99]. When the concentration ratio (HF/H_2_O_2_) is low (from 1.5:1 to 2.5:1–3:1), etching results in a vertical orientation of the nanostructures for (100)-silicon wafers and an slanted (angular) orientation for (111)-silicon wafers [130]. Since the vertical alignment of the nanostructure allows the molecules of the analyte to easily reach the hot spots, (100) silicon wafers are preferably chosen.

## 4. The Electromagnetic Mechanism of SERS Enhancement with Black Silicon-Based Substrates: Simulations

The main mechanism underlying SERS with black silicon coated with noble metal nanostructures is the SPR, which defines an enhancement of the local electromagnetic field near the surface of the plasmonic nanoparticle. However, when considering black silicon SERS substrates, it is not only the excitation wavelength, type of metal (gold, silver, copper, etc.) and the shape (spherical, star-shaped, etc.) of the nanoparticle that play a role [26]. Other crucial features that determine the efficiency of SERS include (i) the type of black silicon coating (separated nanoparticles, nano-islands, pseudo- or continuous layer) [17,116], (ii) the type of geometry of the black silicon supporting the plasmonic structures (holes, pores, pillars, cones) [69,73,120], (iii) the silicon core of the plasmonic structures [17,18] and (iv) the presence or absence of interlayers between the metal structures and the silicon core [122,125]. In this section, the effects of the aforementioned properties on SERS with black silicon-based substrates and the corresponding simulation studies supporting the experimental results are reviewed with respect to the main types of black silicon geometries. However, we will not discuss the phenomenon of SPR, as the relationship between SERS and SPR is discussed in detail in, e.g., reviews [131,132,133].

**Figure 7:**
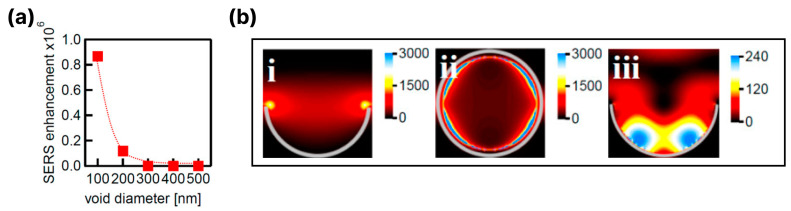
Local field enhancement in pores and Raman signal enhancement: (**a**) SERRS enhancement in the enhanced field region on voids for increasing void diameters; (**b**) field intensity distribution (|E|^2^) in the void for a 100 nm cavity: (i) cross section and (ii) the view from the top. The field is concentrated in a small volume near the rim. (iii) Intensity in a 200 nm cavity with an additional mode deeper inside of the void (the volume mode) [91]. Adapted with permission from Ref. [91] Copyright © 2024, American Chemical Society.

As mentioned above, black silicon produced using electrochemical etching techniques mainly has a porous structure, and the metallization of porous black silicon takes the form of a continuous layer with a thickness in the nanometer range [71]. It is assumed that in such a coating, the metallized pores mainly contribute to the amplification of the Raman signal. In [91], it was shown that the arrays of aluminum nanovoids provide reproducible SERS in the deep UV. The resonant SERS measurements were performed using arrays of cavities with diameters ranging from 100 nm to 500 nm and adenine solution (1 mM) as a testing analyte. The authors showed that the maximum Raman signal enhancement occurs with 100–200 nm voids and decreases with a decreasing diameter (see Figure 7a). The finite-difference time-domain (FDTD) simulations allowed a mechanism for such a behavior to be proposed: in 100 nm aluminum voids, the local field enhancement occurs in a small volume at the edge of the pore (Figure 7b(i,ii)), while in 200 nm voids, the local field is small at the edge but an additional plasmon mode occurs at the bottom of the cavity (see Figure 7b(iii)). Thus, the strong localization of the field in 100 nm pores leads to a significant enhancement of the Raman signal of the molecules adsorbed at the edges, while in larger pores, the SERS enhancement mainly originates from the molecules reaching the bottom of the pore.

**Figure 8:**
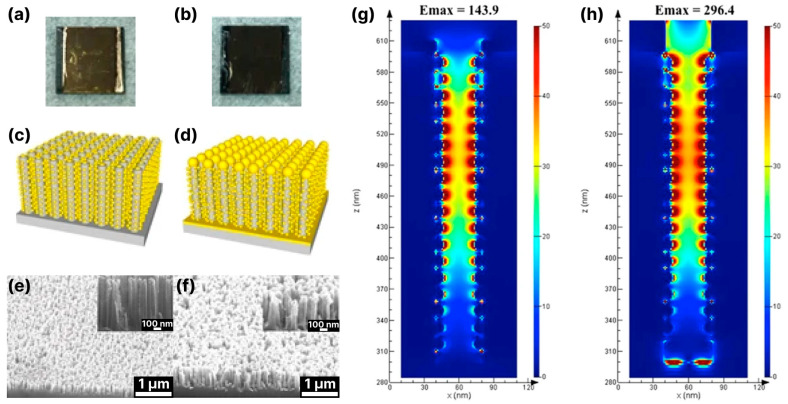
The SiNW arrays decorated with Au NPs and electromagnetic field enhancement: (**a**) photograph of the SiNW arrays with Au NPs decorated sidewall via the OAD process; (**b**) photograph of the SiNW arrays with Au NPs and Au metal backplate (AuMBP) as the desired SERS substrate; (**c**,**d**) are the 3D model corresponding to (**a**,**b**), respectively; (**e**,**f**) are the SEM images corresponding to (**a**,**b**), respectively; (**g**) FDTD simulation results of the electrical field intensity for a control group (SERS substrate composed of SiNW arrays with Au NPs); (**h**) Trail #8 SERS substrate (SERS substrate composed of SiNW arrays with Au NPs and AuMBP). The diameter of Au NPs on the sidewall is set as 13 nm, the distance between SiNW arrays is set as 40 nm and the thickness of AuMBP at the bottom is set as 20 nm [75]. Adopted from [75].

In the case of black silicon fabricated with MACE, silicon nanowires are often produced. As already mentioned, these SiNWs are refined with noble metal NPs as a result of one-step MACE and can be left as they are, but the remaining NPs are often removed with HNO_3_, and then, SiNWs are additionally metallized. In [75], the key difference between these two approaches was shown with respect to the enhancement mechanisms responsible for SERS. In the as-prepared SiMWs decorated with metal NPs, if they are localized on the sidewalls of the SiNWs and some are distributed at the bottom between the SiNWs, additional metallization can lead to the formation of a so-called metal backplane [67,67] or an additional metal layer around the bottom of the SiNWs. It is supposed that this backplane can amplify the Raman signal by (i) preventing additional reflections of the backscattered field from being absorbed by the black silicon, (ii) creating additional hot spots around the SiNWs and (iii) creating the conditions for a coupling effect that leads to the formation of resonating nanoantenna arrays. These resonating nanoantennas are, in fact, the metal nanoparticles supported by the SiNWs and the metal backplane (or BARNA—Backplane-Assisted Resonating Nanoantenna Array) [67]. As can be seen in Figure 8 [75], the addition of the metal backplane results in a two-fold increase in the local electric field (Figure 8b) and stronger backscattering, which favors plasmonic resonances in the nanoparticles at the sidewalls when excited with a 785 nm laser source. This increases the number of hot spots, which are mainly located between the NPs. The simulation results were fully supported by the experiment with thiophenol molecules SAM, where a significant increase in the Raman intensity of the characteristic band 1073 cm^−1^ was achieved with backplane SiNWs compared to Au NP-decorated SiNWs. It has also been shown that in SiNWs arrays and arrays with single nanowires grouped in dimers or trimers, where the distance between the SiNWs is about twice the size of a metal NP, the hot-spot region is not localized at the top edges of the NWs but at the bottom between the NWs [134], and the accessibility of this region to the metal NPs is a prerequisite for good Raman signal enhancement.

However, it is not always possible to introduce the NPs between the SiNWs or to produce a metal backplane. The poor wettability of the SiNWs prevents the Ag- or Au-containing solutions from getting between the SiNWs. This leads to the formation of metallic dendrites mainly at the SiNWs tips, as shown in [76]. In such cases, the Raman signal enhancement is mainly determined by the morphology of the dendrites, and the hot spots are localized in the narrow gaps, intersecting stems, sharp branches and between NPs that are close to each other and form these dendrites. As a result, SiNWs only provide surface roughness and are otherwise not involved in the amplification of the Raman signal.

**Figure 9:**
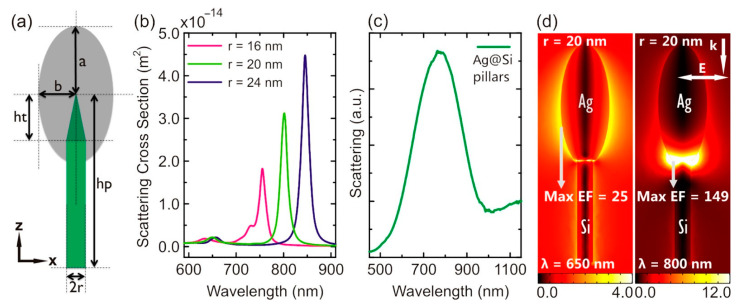
(**a**) Schematic picture of an Ag NP in the x–z plane. (**b**) Calculated scattering spectra of isolated Ag NPs with a varying Si pillar radius, r. The Ag NP parameters used were a = 155 nm, b = 62 nm, ht = 100 nm and hp = 400 nm. The tips at the bottom of the Ag ellipsoid are rounded (5 nm in radius). The normal incident light is polarized along the *x*-axis. The calculations were performed using FEM. (**c**) Measured scattering spectrum of Ag NPs with r ≈ 20 ± 4 nm, hp ≈ 400 nm and pillar density ρ_NP_ ≈ 18 ± 2 pillars/μm^2^. The thickness of the e-beam evaporated Ag metal film is 200 nm. (**d**) The calculated E-field enhancement distribution (|E|/|E0|) around a single Ag NP for the LSPR peaks at λ = 650 and 800 nm. The Ag NP parameters used were the same as in part b for r = 20 nm. The color map range for the cavity LSPR at λ = 800 nm was enhanced for clarity [135]. Reprinted with permission from Ref. [135] Copyright © 2024, American Chemical Society.

The situation changes drastically for ICP-RIE-fabricated silicon structures of different geometries when the silicon core starts to contribute to the amplification of the Raman signal. In [135], Ag-covered nanopillar arrays were shown to exhibit significant Raman signal enhancement when used for the analysis of trans-1,2-bis(4-pyridyl)ethylene (BPE). Simulations using the finite element method (FEM) made it possible to explain the experimental observations. The simulations showed that there are two localized SPR peaks (LSPR) at 650 nm and 800 nm, which determine the amplification of the Raman signal. The localized field at 650 nm has a clear dipolar pattern, and the dominant enhancement occurs at the sidewalls of the Ag NPs, while at 800 nm the electric field is significantly enhanced at the bottom of the metal cap in the region of the contact between the silicon core and the silver NP (see Figure 9). The size of the capping NPs and the distance between them, as well as the ratio of the metal-ellipsoid parameters, influences the Raman signal enhancement and can shift the position of the LSPRs. This partly explains the broadening of the measured scattering spectrum compared to the simulated spectrum. An obvious advantage of such a substrate is the coincidence of the LSPRs with the widely available 633 nm and 785 nm excitation laser sources.

**Figure 10:**
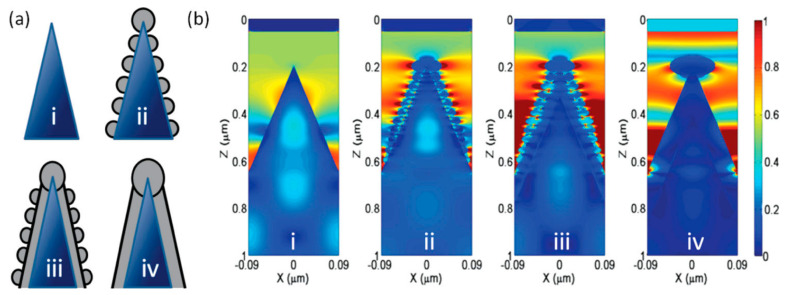
(**a**) Schematic configuration of different Ag-coated BSs. (**b**) The corresponding simulated electric field distributions for the different cases. The color map is normalized for the convenience of the comparison [17]. Reprinted with permission from Ref. [17] Copyright © 2024 John Wiley and Sons.

Cone and pyramidal types of black silicon offer more rigid and less fragile structures than nanopillar ones. In contrast to the porous/pillar/NW types of black silicon, more areas on the sidewalls and bottom of the structures are accessible to the analyte molecules [17,18,121]. The metal coating of the cone-shaped structures is more diverse and ranges from continuous and thick layers [69,79], metal flakes [81] and nano-island [119] to spatially separated nanoparticles [118,124,125]. Different types of metal coatings and their thicknesses define different schemes of electromagnetic field enhancement and the contribution of the silicon substrate to this enhancement. The thickness of the sputtered metal layer determines the geometry of the metal structures [17]: 35–55 nm of Ag coating leads to separated NPs distributed on the silicon cone, 80–100 nm-to-thick films with clearly resolved NPs and 150 nm-to-thick continuous Ag films. Structures with separated Ag NPs (35–55 nm) show good Raman signal enhancement, which increases at a 80–100 nm strongly roughened film but decreases significantly at an Ag film thickness >150 nm. With the FDTD simulation of the possible structures (see Figure 10a), it could be shown that even without an Ag coating (Figure 10(i)), a local field enhancement occurs but mainly in the corners of the neighboring cones due to the light-trapping effect (see Figure 10b). The coupling of the incident light to the LSPs of the separated Ag NPs occurs in separated NPs (Figure 10(ii)) and in metal-bridged NPs (Figure 10(iii)), but the penetration of the light into the interior of the silicon cone in this case (Figure 10(ii)) weakens the coupling effect and the resulting enhancement. The metal-bridged structures (Figure 10(iii)) show much stronger field enhancement and more hot spots than in the other cases. Coupling with a continuous Ag film also allows for a strong enhancement and probably resembles, to some extent, the enhancement effect supported by the backplane [67], but the number of hot spots is lower compared to the structures (Figure 10(iii)), and the accessibility of the cavities between the cones is worse, which explains the lower experimental Raman signal enhancement compared to the other structures (except the bare silicon cones).

**Figure 11:**
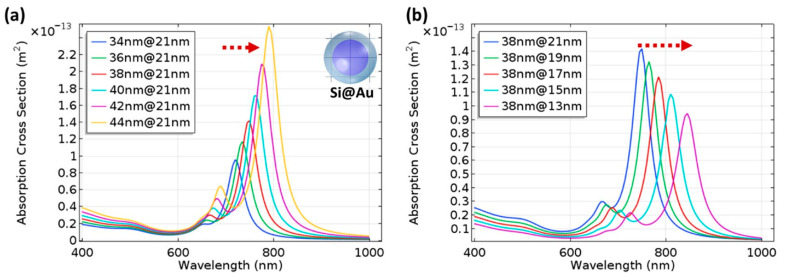
Absorption cross section as a function of wavelength simulated for an Si@Au spherical nanoparticle. (**a**) Changing the Si-core radius from 34 to 44 nm at an Au-shell thickness of 21 nm, (**b**) changing the Au-shell thickness from 13 to 21 nm at a Si-core radius of 38 nm [18]. The excitation wavelength was λ_ex_ = 785 nm. The red arrows indicate the direction of the shift of the LSPR peak position with (**a**) the increase of the silicon core and (**b**) the decrease of the Au layer thickness. Adapted with permission from Ref. [18] Copyright © 2024, American Chemical Society.

The high contribution of metal bridges between the metallic NPs formed on the sidewalls of the pyramidal black silicon structures and the overlapping of the NPs was demonstrated in, e.g., [18,136]. Through the FEM simulations of the interactions between the overlapping (bi-)spheres and the bridged spherical nanostructures with the incident plane wave, two distinct plasmon resonances were obtained (the wave polarization coincided with the major axis of a complex nanoparticle): a transverse peak localized in the visible spectral region and a longitudinal peak in the NIR. The longitudinal peak surpassed the transverse peak in size, indicating that the local field enhancement is maximized upon excitation in the NIR. Furthermore, the presence of a silicon core in the apex plasmonic structures likely defines the optical response of the Au-coated black silicon structures. Increasing the radius of the silicon core leads to an increase in the magnitude and a red shift of the LSPR peak (see Figure 11a). A similar red shift, albeit with decreasing intensity, can be achieved by thinning the gold layer (see Figure 11b). These results indicate that it is possible to develop black silicon-based SERS substrates that require extremely little noble metals but provide significant local enhancement of the electric field and thus SERS.

Matching the excitation wavelength with the position of the LSPR of the plasmonic structures is crucial. Although a small LSPR is observed near 500–550 nm, the main contribution to the SERS performance originates from the NIR LSPR. In [116], it was experimentally demonstrated that excitation with λ_ex_ = 532 nm elicits SERS, which is, however, much weaker than that for excitation with λ_ex_ = 785 nm, consistent with the LSPR of the described structures. In addition, the sizes of the plasmonic structures are not the same and have a certain distribution, which makes it possible to match the excitation wavelength of λ_ex_ = 785 nm with the LSPR of these particles. Similar results were obtained with RIE-fabricated silicon structures capped with gold with probing analytes R6G and MG [123]: excitation with λ_ex_ = 532 nm led to lower Raman signal enhancement than excitation with 633 nm and 785 nm.

**Figure 12:**
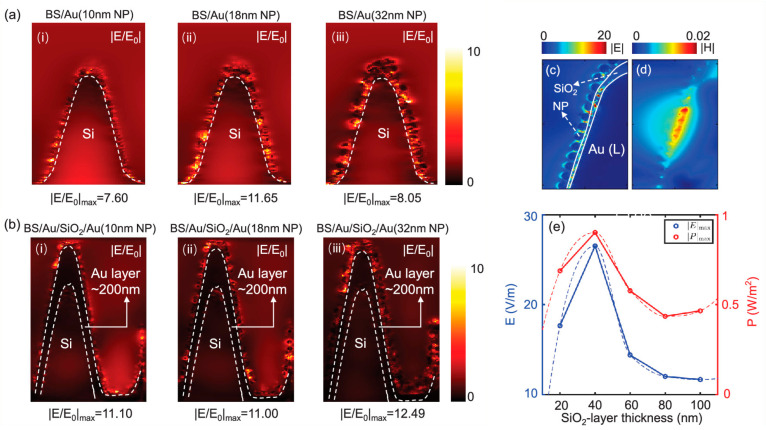
Local electric field distributions on selected cross sections of a bare (**a**) and layer-coated (**b**) BS cone with different sizes of NPs. The profile of the BS cone and the conformal Au layer are marked by dashed white curves. (i), (ii) and (iii) correspond to the coating of the structures with NPs of sizes 10 nm, 18 nm and 32 nm, respectively. (**c**,**d**) Local electric and magnetic field distributions on a selected part of the Si cone in the BS/Au(200 nm-L)/SiO_2_/Au(60 nm NP) structure. (**e**) Dependence of the maximum amplitudes of the localized electric field and the energy flux density on the thickness of SiO_2_ layer, fitted by both linear and piecewise cubic Hermite curves, indicating an optimized value of SiO_2_ thickness close to 40 nm [122]. Adapted with permission from Ref. [122] Copyright © 2024, American Chemical Society.

Coating the black silicon substrate with NPs or continuous or pseudo-continuous layers is now almost a traditional approach. There are various variations of this, e.g., by introducing additional layers on the silicon structures. Some layers are aimed to provide better adhesion of noble metal layer on the silicon surface [17,119] or to endow with additional properties [125]. For example, additional coverage with graphene oxide layers of the black silicon initially coated with Ag NPs results in better Ag NP stability and prevents their oxidation upon exposure to oxygen [125]. In [122], a thin (tenths of nanometers) layer of SiO_2_ between the continuous Au layer and Au NPs distributed atop is introduced as a dielectric spacer, which favors the formation of single subwavelength nanocavities in it. The ensemble of nanocavities is shown to be beneficial in electromagnetic field enhancement. The formation of Au and SiO_2_ layers significantly improves Raman signal enhancement, with the former acting as a reflector and preventing light absorption by the silicon core and the latter serving as a volume for confining the electric field in nanocavities resulting from the hybridization of the electromagnetic field scattered by the NPs and the electromagnetic field reflected by the Au layer (see Figure 12). Such black silicon/Au layer/SiO_2_/Au NPs provides better Raman signal enhancement than black silicon coated with nanoparticles, and the performance of such a layered system can be improved by choosing the appropriate thickness of the layers. For the analyzed structures, these thicknesses were 200 nm for the Au layer and 40 nm for SiO_2_ (see Figure 12, right).

## 5. Biosensing with Black Silicon-Based SERS Substrates

Biosensing, biomedical and bioanalytical applications place several stringent requirements on the materials that can be used for bio-applications. Non-invasiveness, the ability to perform analysis remotely, speed, selectivity and sensitivity are among the key requirements [137]. The possibility of making chemical modifications to the working surfaces to customize selectivity to specific biomolecules is also important [138,139]. The SERS technique enables a variety of bio-applications, from single molecule detection [140,141] to in vitro [142] and in vivo [143] studies of cells and tissues. One of the main advantages of black silicon as a substrate for SERS biosensing is that the key properties that determine the applicability of the material for bio-applications can be tailored to the desired properties over a wide range by varying the fabrication and metallization parameters.

Another prerequisite for biosensor technology is the preferred use of NIR light sources for excitation. The reason for this is that NIR laser irradiation is less harmful to biological objects than irradiation in the UV-to-green spectral range [144] and that it can avoid autofluorescence of bio-samples [20]. This requirement is automatically fulfilled when using noble metal-coated black silicon, as the main maximum of the plasmon resonance is in the NIR range and its position can be regulated by varying the shape of the silicon structures and the thickness of the metal layer, as already mentioned above.

Nevertheless, most SERS studies were performed with black silicon substrates using standard test molecules, such as the dyes methylene blue [94], rose bengal [77], rhodamine 6G (R6G) [17,69,118,124,125], etc., 4-mercaptobenzoic acid (4-MBA) self-assembling monolayers (SAM) [18,116] and Cu(II)-tetrakis(4-N-methylpyridyl) porphyrin (CuTMpyP4) [71]. These proof-of-concept studies showed high enhancement factors of up to 10^10^ and detection limits of 10^−15^ M (see Table 1 and Table 2), which definitely indicates a high potential of black silicon for real-world applications, especially considering its availability, low cost and ease of fabrication, as well as the possibility of scaling up to an industrial scale.

SERS studies on real biological objects, such as bio-macromolecules, cells and tissues with black silicon, are not as widely represented as studies with test molecules. In [67], the detection of the vitamins riboflavin and thiamine at concentrations of 10^−7^ M and two types of single-stranded DNA at concentrations of 10^−9^ M was performed with the optimized BARNA black silicon substrate. Detection of one of the most commonly used antibiotics, tetracycline, was demonstrated [80], showing the potential of using black silicon SERS biosensors for monitoring drug levels in blood and disease progression.

Black silicon provides a rigid and stable substrate that is inert to biological samples and offers significant amplification of the Raman signal, which is crucial for biosensing. In [123], a proof-of-concept experiment was performed to demonstrate the ability to detect 3–30 kDa proteins isolated from blood plasma, although no detailed analysis of the spectra was provided. Nevertheless, such a demonstration shows the potential of black silicon for clinical use as it enables the analysis of biofluids.

Porous silicon covered with Ag NPs was shown to be able to detect bovine serum albumin (BSA) at concentrations ranging from 0.5 mg/mL to 2.0 mg/mL and normal serum [145]. However, bare porous (black) silicon showed virtually no SERS signal from BSA tested in the same concentration range.

**Figure 13:**
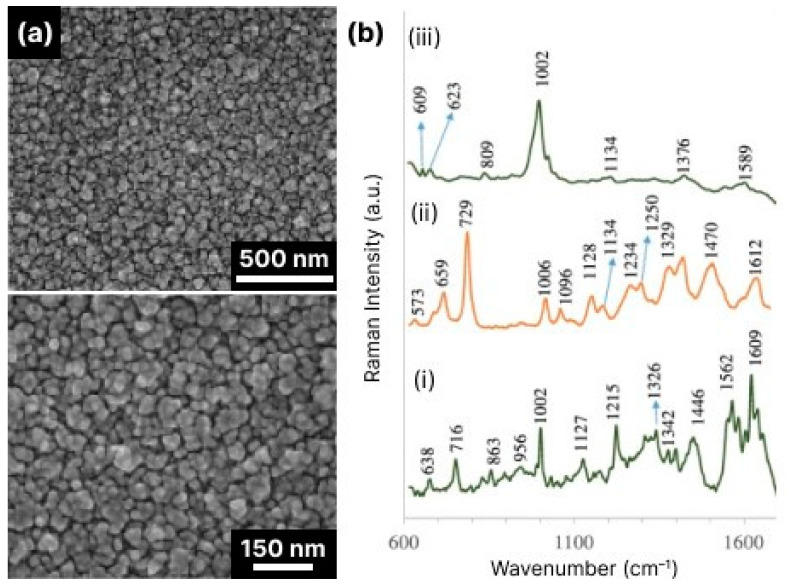
Optimized gold-capped porous silicon for human biofluid analysis: (**a**) SEM micrograph of porous silicon electrochemically etched in 100% butanol solvent and covered with a 30 nm gold layer; (**b**) average SERS spectra of (i) whole human blood, (ii) cerebrospinal fluids and (iii) urine obtained onto an optimized SERS-active Au/Si surface. Mean spectra were averaged over 30 spectra from different spots. Experimental conditions: 5 mW of 785 nm excitation, 4 × 10 s acquisition time. The SERS spectra have been baseline corrected and shifted vertically for better visualization [92]. Adopted with permission from Ref. [92] Copyright © 2024, Elsevier B.V.

Black silicon, produced via electrochemical etching and sputtered with gold in thicknesses ranging from 10 to 300 nm, enabled the label-free analysis of human biofluids, including whole blood plasma, urine and cerebrospinal fluid (CSF) [92] (see Figure 13). The best substrate SERS performance in this case was obtained with NIR excitation and a rough Au layer thickness of 30–50 nm, which is consistent with the theoretically predicted best operating conditions (see Section 3). The optimized gold-covered porous black silicon was used to determine the concentration (down to nanomolar) of neopterin—a marker for bacterial infection—in cerebrospinal fluid infected with *Neisseria meningitidis*. The latter suggests that some optimized versions of black silicon for SERS are almost ready for clinical trials.

Porous silicon disks, produced using a more complex method combining photolithography and electrochemical etching [146], with Ag NPs reductively synthesized and aggregated inside the pores, were applied for glutathione (GSH) detection in biofluids. Glutathione is one of the key participants of the human antioxidant system, and its altered levels may indicate the progression of diverse diseases, including Alzheimer’s disease [147], cancer [148], etc. In order to increase the sensitivity of the substrate to GSH as compared to other biothiols, such as cysteine and homocysteine, the authors carried out additional modification of the Ag NP surface with 5,5′-dithio-bis(2-nitrobenzoic acid) (DTNB) which, under particular measurement conditions, enables the selective detection of GSH with an LOD around 75 nM.

As already mentioned, micro-structured silicon is not always referred to as black silicon in the literature, and in principle, it is not always black silicon, as it still retains a noticeable reflectivity. However, despite the deterioration in light trapping, such structured silicon also shows quite good SERS, which can sometimes be combined with other detection methods. For example, micro-/nano-structured silicon [149] showed the potential to determine blood glucose levels via SERS and through surface-enhanced fluorescence (SEF) with an LOD of 10^−9^ M for SERS and 10^−6^ M for SEF and SERS EF around 10^6^. The demonstrated LOD surpasses the LOD estimated for Ag NP-modified hydrogel microspheres (10 μM) [150], self-assembled Ag NPs (10 nM) [151] and star-like Au NPs (10^−7^ M) and repeats the LOD of albumin-modified star-like Au NPs (10^−9^ M) [152], although it is lower than the glucose LOD of micro-structured silver substrates [153], which reaches the concentration of 0.5 amol/L in deionized water.

It is also important to note that such a phenomenon as a plasmon-driven reduction-oxidation (redox) process (a process that does not require additional catalysts) [154] should be considered when metal-coated black silicon-based SERS substrates are used for bioanalytical studies. Plasmon-driven redox processes occur when SPs serve as catalysts, i.e., “hot electrons” can be transferred to the excited state of the adsorbed molecules of the analyte to provide the activation energy required for the reaction [155]. Such plasmon-driven redox reactions occur in both atmospheric and aqueous environments and can significantly affect the structure of the biomacromolecules and make the results unreliable. However, black silicon substrates offer several ways to avoid such problems. One of the approaches was proposed in [81] and consists of using bare black silicon prepared with ICP-RIE directly for SERS. As mentioned earlier, even bare pyramidal black silicon contributes to SERS due to localized electromagnetic field enhancement in the voids between the cones [17]. For example, such metal-free enhancement was also demonstrated with porous black silicon with a detection limit of about 10^−8^ M for R6G solutions [70]. It has been shown that bare black silicon allows for the detection of *para*-aminothiophenol at concentrations as low as 10^−6^ M without this molecule being converted to 4,4′-dimercaptoazobenzene by plasmons, which is often the case in measurements with gold-coated black silicon [81]. The selection of suitable measurement conditions can also enable the non-perturbing SERS detection of organic molecules. In [121], the detection of 4-MBA SAM was demonstrated by varying the irradiation intensity towards the substrate without damaging the molecule through its decarboxylation. The main disadvantage of such an approach is the need to perform additional analyses before the main measurements to determine “safe” measurement conditions.

SERS studies on living cells can make an important contribution to the development of new classes of drugs and medicines and to the identification of molecular mechanisms that determine the progression of various diseases. The use of black silicon-based SERS substrates for living cell analysis is subject to several limitations. For example, porous black silicon obtained via electrochemical anodization requires additional layers to protect the surface from hydrolytic attack in aqueous solutions, as this leads to degradation of the surface [90]. The rate of such degradation in biomimetic liquids can be up to 9 μm/day [156]. The thinner the silicon structures or the higher the porosity of the black silicon, the less stable and durable the SERS substrate based on black silicon is. Since the measurements on living cells require the sample to be measured in a liquid medium, black silicon substrates produced via electrochemical etching or MACE are difficult to use.

**Figure 14:**
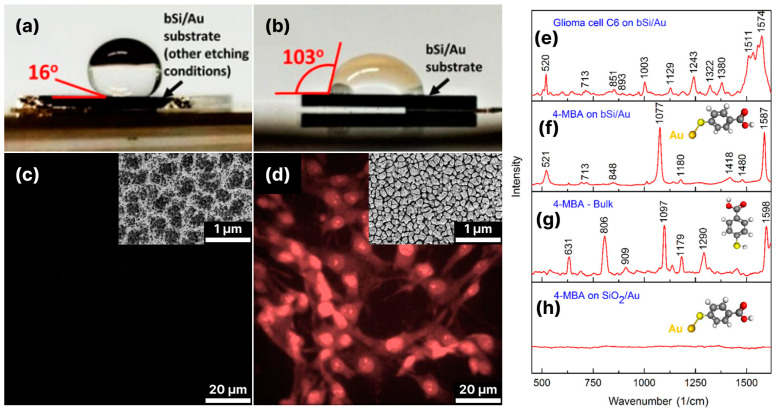
Black silicon substrates applicable and non-applicable for living cell analysis: water-drop hydrophilicity assay of (**a**) Au-coated lace-shaped black silicon and (**b**) Au-coated pyramid-shaped black silicon; viability assay performed for cells grown on (**c**) a hydrophobic lace-shaped black silicon (no adherent cells) and (**d**) hydrophilic Au-coated pyramid-shaped black silicon (dense monolayer of cells); comparison of the Raman spectra of the 4-MBA monolayer on the SiO_2_/Au smooth substrate (**h**) of bulk 4-MBA (**g**), and SERS spectra of 4-MBA (**f**) and living rat glioma cells (**e**) on the Au-coated pyramid-shaped black silicon substrate. The spectrum of a living cell was recorded in an aqueous Hepes-buffer solution. The viability assay included cell fixation, permeabilization and staining with propidium iodide fluorescent dye to visualize cells grown on the Au-coated black silicon substrate. The insets in (**c**,**d**) are SEM images of the corresponding structures. The excitation wavelength was 785 nm [18]. Adapted with permission from Ref. [18] Copyright © 2024, American Chemical Society. Insert image in (**c**) adapted from [121].

Another obvious limitation is the hydrophobicity of the as-fabricated black silicon. As mentioned above, hydrophobicity can originate from surface modification during the fabrication process and be caused by the non-polar groups, such as the result of wet etching, or due to the nano-/micro-structuring of the surface. Both reasons can be overcome by producing black silicon through free-mask ICP-RIE: (i) the masking layer that is formed in a mixed-mode ICP-RIE desorbs upon heating and black silicon is only silicon [112,114], and (ii) the shape of the silicon structures, their size and density can by controlled by varying the etching conditions [111]. In [18,80] it was demonstrated that the pyramidal (conical) and cylindrical black silicon coated with gold is hydrophilic, while either its salinization [80] or a change in the ICP-RIE conditions (switch from cryogenic to RT) [18] resulting in sharp lace-like structures makes black silicon hydrophobic (see Figure 14).

Nonetheless, the hydrophobicity of the black silicon can still be a useful property as it favors self-concentration of the analyte molecules in a limited area upon drying, while a hydrophilic surface favors the spread of the liquid over larger areas resulting in a concentration decrease [80,157].

Several studies have demonstrated the bactericidal effect of black silicon, which is mainly caused by the mechanical contact of bacteria and sharp silicon structures [158,159]. This seems to be a serious limitation for the use of black silica in cellular research. However, the interaction of living cells with micro- and nanostructured surfaces is a complex process that depends on many factors, which can change the properties of materials from bactericidal and unsuitable for working with living cells to fully biocompatible. The spacing between the silicon structures is crucial for bacterial survival: black silicon with large distances between the structures (800 and 1400 nm) demonstrate no bactericidal properties, while 300 nm distances result in high bactericidal efficacy [160]. Moreover, the adhesion of living cells to surfaces is governed by the macro-, micro- and nano roughness of the structures. For instance, the biocompatibility of cone-shaped black silicon outperformed that of traditionally used materials for cell growth (see Figure 14b,d) [48], while hydrophobic lace-shaped black silicon failed as a surface for living cell adhesion and growth (see Figure 14a,c) [18].

**Figure 15:**
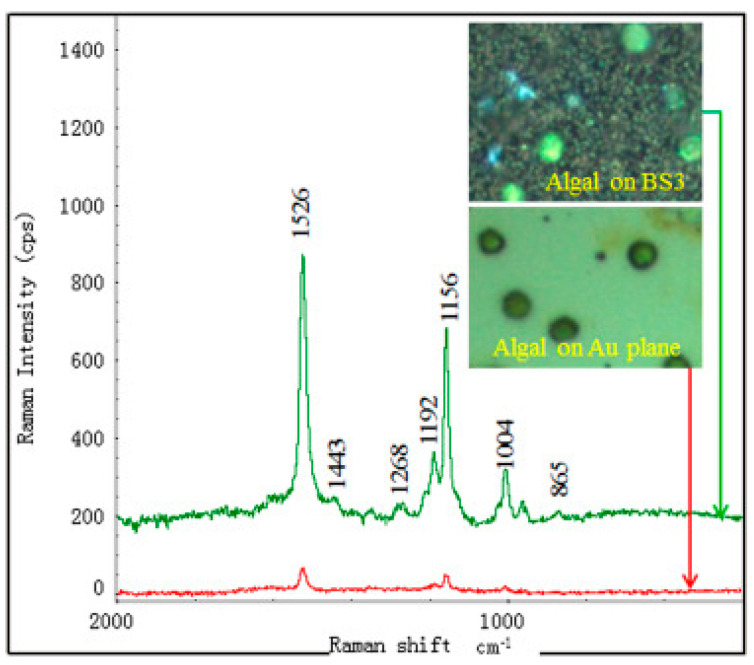
SERS detection of a single algal cell by using the BS3 substrate deposited with a 400 nm gold layer. The insets are the images of the algal cells dispersed at the substrates taken from the optical microscope [79]. Reprinted with permission from Ref. [79] Copyright © 2024, Elsevier B.V.

One of the first mentions of SERS of living cellular organisms was the measurement of a single algal cell *Chlorella vulgaris* [79] with cryogenic ICP-RIE black silicon coated with a 400 nm thick gold layer. The bands assigned to carotenoids, proteins, saturated/unsaturated lipids and phospholipids were clearly distinguished (see Figure 15). The laser with λ_ex_ = 532 nm was used to obtain the resonance scattering from the cells. However, the excitation in the visible range is not optimal to achieve both the SPR of black silicon coated with gold and the viability of the cells. The latter was not additionally controlled. However, the high thickness of the gold layer nullified the role of black silicon in signal amplification and micro-structuring—a confluence of circumstances that allowed measurements of a living object.

Later, gold-coated black silicon fabricated via ICP-RIE was applied for bacterial detection [161]; however, a high level of SERS spectra variability was observed, which is more likely due to the difference in the distances between the probed molecules and the SERS surface.

The hydrophilicity of the pyramidal black silicon coated with gold defined its outstanding biocompatibility and, thus, applicability for living cells analysis (see Figure 14d) [18]. It was also favored by a high enhancement factor (>10^8^) demonstrated by the structures of such types with 4-MBA SAM (see Figure 14f–h). It allowed the SERS spectra of rat glioma cells sub-cultured on the black silicon coated with a 50–80 nm nano-rough gold layer to be obtained (see Figure 14e). The characteristic bands of nucleic acids (e.g., 894 cm^−1^−adenine), proteins (e.g., 1003 cm^−1^–phenylalanine symmetric ring breathing; 1243, 1273, 1288 cm^−1^ assigned to amide III) and lipids (e.g., 1380 cm^−1^−CH_3_ symmetric stretch) in the “fingerprint” region of the SERS spectrum of cells are clearly distinguished.

**Figure 16:**
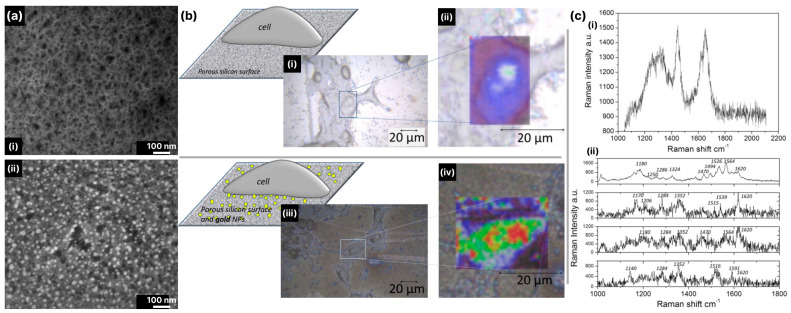
MCF breast cancer cells grown on bare mesoporous silicon with and without decoration with Au NPs: (**a**) SEM micrographs of (i) bare and (ii) Au-NP-decorated silicon structures; (**b**) optical images of cells grown on (i) bare and (iii) Au-NP-decorated silicon structures and Raman and SERS maps of cells on bare (ii) and (iv) Au-NP-decorated silicon structures; (**c**) Raman and SERS spectra of cells grown on (i) bare and (ii) Au-NP-decorated silicon structures. Raman and SERS maps were reconstructed using the band at 1445 cm^−1^ assigned to CH vibrations. The laser power was 3 mW and 12 μW for spectral mapping on bare and Au-NP-decorated mesoporous silicon, respectively. Measurements were carried out with a laser with λ_ex_ = 633 nm [93]. Adopted with permission from Ref. [93] Copyright © 2024, Elsevier B.V.

Good cell adhesion properties of gold-decorated mesoporous silicon produced via anodization were demonstrated in [93] (see Figure 16). The roughness of the substrate favored good adhesion and he proliferation of MCF-7 breast cancer cells, although no test of cell viability or comparison with standard materials for cell culture was performed, and the possible cytotoxic effects may still be of concern. It was shown that with bare mesoporous silicon substrate, the spectral features of amide III, amide I and CH vibrations could be detected (Figure 16b(i,ii) and Figure 16c(i)), while decorating the silicon with Au NPs provided the opportunity to visualize the distribution of various adhesion molecules, such as integrins, on the substrate (Figure 16b(iii,iv) and Figure 16c(ii)). In contrast to [18], where the SERS measurements were performed on living cells, this study was performed on cells fixed with formalin, which can lead to a change in cell structures and incorrect assumptions and must therefore be viewed critically when formulating conclusions.

The regularity of the silicon structures enables a very uniform distribution of noble metal nanoparticles and thus the mapping of cells with a high spatial resolution, which is improved by supplementing the density of the silicon structures with the density of the “hot spots” on each individual structure. The high density of “hot spots” and their uniformity is also crucial when the accumulation of multiple spectra of a sample is required, such as heterogeneous bioliquids containing multiple biomarkers. In this context, high-density 3D microstructures with similar light-trapping properties but different origins (non-silicon) have also gained interest as SERS-active substrates for bio-sensing and cell analysis. The platinum black (Pt-black) substrate is one of these materials [162]. Pt-black has been shown to successfully detect exosomes—vesicles secreted by cells that serve as biomarkers for various diseases—and distinguish between cancer-derived and normal fibroblast-derived exosomes with high sensitivity and selectivity.

Interestingly, the number of SERS studies on living cells with black silicon substrates is still very limited, although the exploration of cell structures inside the cells with plasmonic nanoparticles [163,164,165] or cell membranes with hybrid flat surfaces (e.g., multilayer metal-insulator-metal nanolaminated SERS substrates [166], reduced GO sandwiched by silver and gold nanostructures on flat silicon [167], etc.) is rapidly developing. The lack of attention to black silicon as a material for bioresearch is probably since its antibacterial properties were first demonstrated, while attention to the biocompatible modifications of black silicon has only recently been aroused. Moreover, the instability of the solutions of plasmonic NPs, their fast degradation and agglomeration are obvious obstacles to their bio-applications. Black silicon-based substrates, in turn, surpass the NP-based approaches due to the stability of black silicon, inertness and the possibility of long-term use. For example, black silicon fabricated with MACE and coated with Au NPs showed a good enhancement factor of 10^6^ and remarkable SERS performance for almost 120 days of storage [105]. As mentioned above, the GO coating of a black silicon-based SERS substrate prolongs the shelf life by preventing oxidation [125]. Black silicon prepared with RT ICP-RIE and coated with a nm-thick gold layer was found to be stable and showed significant Raman signal enhancement for more than 20 months of storage [121]. In addition, the possibility of reusing this substrate through purification with oxygen plasma was demonstrated for both molecules covalently bound to gold (4-MBA) and non-covalently adsorbed molecules (medical drug doxorubicin) with about ten possible cycles of reuse.

**Figure 17:**
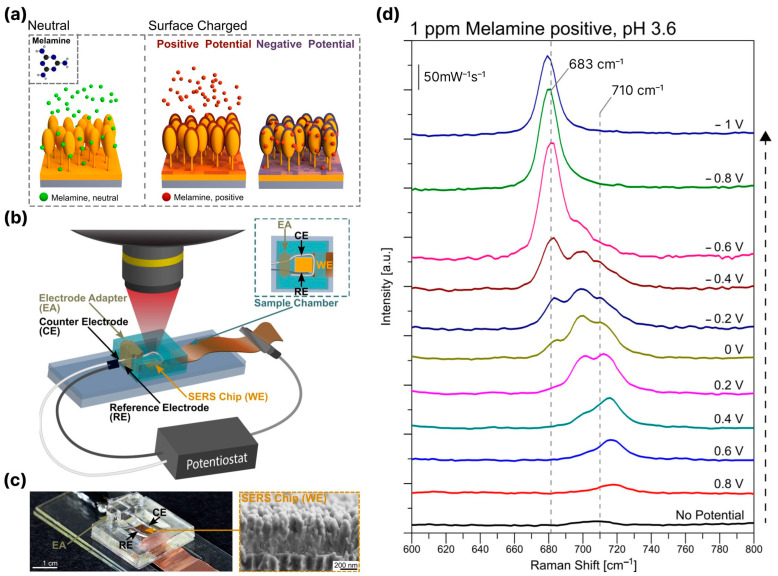
(**a**) Working principle of the electrochemically assisted SERS-based detection showing the variations in surface charges on the Au-capped nanopillar SERS substrates and the suggested interaction of the surface with melamine. (**b**) Illustration of the custom-made electrochemical-SERS platform and its respective system interfacing. (**c**) Photo of the assembled detection chamber and SEM image of Au-capped nanopillar structures for SERS detection. (**d**) Comparison of neutral and positively charged melamine in PBS at no (black line) and −0.8 V vs. Ag/AgCl (green line) applied potential. SERS spectra of 1 ppm melamine in PBS at pH 3.6 dependent on the applied potential [171]. Adapted with permission from [171]. Copyright © 2024, American Chemical Society.

Another perspective application for black silicon-based substrates is electrochemical SERS, which consists of SERS measurements of molecules of the analyte adsorbed on the rough surface of the noble metal electrode, for which the potential can be varied, thereby controlling the surface charge of the electrode [168]. The electrodes are often electrochemically roughened. Such micro/nano-structuring of the electrode surface is random and uncontrollable, which makes it difficult to achieve the required Raman signal enhancement and severely limits the reproducibility of the measurements [169,170]. Metal-coated black silicon offers an excellent substitute for traditional electrodes. In [171], Au-capped black silicon with a pillar geometry was successfully used for real-time electrochemical tests to detect the milk adulterant melamine. Changing the potential applied to the Au-capped black silicon enables either the adsorption of the melamine molecules (negative potential), resulting in relevant SERS spectra, or their desorption (see Figure 17a). Using a single chip electrochemical SERS platform (see Figure 17b,c), it was possible to detect melamine contamination in the range of applied potentials (−1.0; 0.8) V with detection limits of 0.01 ppm in phosphate buffer solutions and 0.3 ppm in milk.

Electrochemical applications of black silicon can also make an important contribution to the study of the behavior of molecules at the solid–solution interface (electrode). This can provide valuable information on the conformational changes of biomacromolecules, such as proteins or DNA, induced by different surface charges and shed light on the mechanisms of ligand–receptor interactions or interactions with biomembranes [168] determined by the changes in transmembrane potentials [172].

## 6. Conclusions

The SERS technique occupies an important place in research, from fundamental science to practical application. Nowadays, it is impossible to imagine biomedicine, forensics, environmental protection, food safety, etc., without the highly sensitive SERS technique. The emergence and progress in the development of numerous SERS-active substrates for analysis and research is helping to confidently consolidate this trend. Black silicon, a micro/nano-structured type of silicon, is a unique material for SERS. Different forms of silicon structures and a variety of methods to fabricate them, multiple approaches to coat black silicon with plasmonic nanostructures ranging from single NPs to metal islands and continuous NP-decorated layers enable the fabrication of SERS substrates that can meet the requirements of any research and technology task. The long history of silicon microelectronics makes black silicon a suitable material for integration into electronic devices, lab-on-chip systems, microfluidics, etc., and forms a promising basis for the development of SERS-based nanobiophotonic sensors. In this context, taking the advantage of the mentioned above, black silicon can be considered a major component of the SERS biosensor due to several reasons: (i) it contributes to SERS enhancement itself, (ii) it provides a 3D matrix for the uniform distribution of plasmonic structures responsible for plasmonic SERS, (iii) it affects the position of LSPR and shifts it to NIR, which favors NIR operation of the biosensor, (iv) it is stable and cost-efficient, (v) simply produced, with a controllable surface geometry, which allows the substrate to be adjusted to specific demands, (vi) it can be hydrophobic for self-concentrating extremely low amounts of analytes or hydrophilic and biocompatible to allow living cell adhesion and tissue measurements and (vii) it can be easily integrated in existing devices. These show that black silicon offers a perspective material for real world applications and the transfer of SERS sensing to practice. The well-established fabrication technology of black silicon enables the efficient, simple, large-scale and cost-effective production of SERS sensors based on black silicon. Numerous proof-of-concept experiments demonstrate that black silicon has a competitive LOD and EF, which is lower than only several single substrates reported by now, which are often characterized by great complexity and high costs. Despite some limitations, such as hydrophobicity or the environmental instability of some porous black silicon, the emergence of new applications, such as in vitro living cell research and electrochemical SERS, suggests that there is still room for further improvement and the refinement of new designs of cheap, scalable and multifunctional sensors.

## Figures and Tables

**Table 2:** Comparison of the parameters and SERS efficiency of the black silicon-based SERS substrates produced via inductively coupled plasma reactive ion etching (ICP-RIE).

Black Silicon Fabrication	Si Wafer Parameters	Black Silicon Morphology, Tip Density	Metal, Thickness, Layer Type	Metal Deposition Method	Enhancement Factor	λ_ex_, nm	Detected Object, Detection Limit	Reference
Maskless ICP–RIE SF_6_/O_2_: 0–150/0–50 sccm, −110 °C, 3–40 min	(100) 6″	Sharp-tip; Height: 200 nm–6 μm, Spacing: 100 nm–2 μm, 1–30 tips/μm^2^	Au, 100–600 nm, bridged gold	e-beam evaporation, 1 Å s^−1^	N/A	532	R6G; *Chlorella vulgaris* 10 fM (R6G)	[79]
Maskless ICP–RIE: SF_6_/O_2_ 35/45 sccm, 1 Pa, 150 W, 15 min; SF_6_/O_2_ 65/44 sccm, 4.7 Pa, 100 W, 1–25 min	(100) p-type	Pyramidal shaped spikes; pillars	Au 200 nm layer, with a molecularly imprinted polymer layer for target molecules	Magnetron sputtering	N/A	785	Tetracycline	[80]
Maskless ICP–RIE: SF_6_/O_2_ 35/45 sccm, 1 Pa, 150 W, 15 min;	(100) 3″	Randomly-arranged spikes; Height 600 ± 150 nm 60 tips/μm^2^	(i) no; (ii) Ag; (iii) Au; 20–200 nm, metal flakes	e-beam evaporation, 1 nm s^−1^	(i) 10^3^ (ii), (iii) N/A	532	*para*-aminothiophenol (i) 10^−6^ M (ii), (iii) N/A	[81]
Maskless ICP–RIE: SF_6_/O_2_ 15/37.5 sccm, 250 mTorr, 150–170 W, 10–20 min	single crystal	Grass-structured: Height: 2–7 μm	Ag 40–150 nm Separate NPs	e-beam evaporation, 1 Å s^−1^	N/A	785	R6G 10^−3^ M	[118]
Cryogenic ICP-RIE: SF_6_/O_2_ 30.5/27.5 sccm, 10 mTorr, 1000 W; SF_6_/O_2_ 40/18 sccm, 20 mTorr, 1000 W	(100) 4″	Pyramid-like	Ag 35–150 nm, Intermediate 4-nm-thick Ti film; Form NPs to continuous layer	e-beam evaporation, 0.35 nm s^−1^	6.8 × 10^9^	532	R6G	[17]
Maskless ICP-RIE SF_6_/O_2_ 65/44 sccm, 35 mTorr, 100 W RIE power, 20 °C electrode, 10 Torr He cooling; 20 min	(100) p-type (boron-doped), 10–20 Ω × cm, 4″	Needle-like (grass-like); Height: 3.5 μm, Cylinder diameter: 40–240 nm	Au 30–300 nm, 300 nm mainly non-continuous layer, 30 nm, continuous for >30 nm, gold nanorods added	Magnetron sputtered	N/A	514, 633	R6G 10^−6^ M	[69]
Advanced Silicon Etching, SF_6_/O_2_	N/A, 4″	Grass-like	Au 400 nm, Nanoislands; Ti and Ti/Pt adhesion layers underneath Au	e-beam deposition (static and sweep modes)	7.6 × 10^7^	785	R6G 2.4 pg	[119]
ICP-RIE SF_6_/O_2_ of 35/45 sccm, 150 W RIE, 1.0 Pa, 15 min	(100) p-type, 3″	Pyramidal pillars: Height/width = 2.3; Height = 279 nm, Tip diameter 16–20 nm	Ag <100 nm, isolated islands; 1–10 nm chromium adhesion layer	Thermal evaporation; 0.1 nm s^−1^, rotating stage	N/A	532, 633	Thiophenol SAM	[120]
Maskless RIE	N/A	Pillars: Width: 50–80 nm, Height: 600–1600 nm, Density: 3.3, 6.2. 8.9, 14.2, 14.8 and 18.0 pillars/μm^2^	Ag capping of pillars, backplane deposition, 100–200 nm	e-beam evaporation	10^11^	785	Thiophenol	[68]
Two-step ICP-RIE: (1) C_4_F_8_ masking, (2) SF_6_/O_2_ etching 10:9 sccm, 18–30 min, 30 mTorr, 20 °C	(100) n-type (p-doped), 1–30 Ω × cm, 2″	Pillars: Height: 639 nm–2.2 μm Thickness: 110–830 nm 0.56–4.33 tips/μm^2^	Au <25 nm Mosaic pseudo-layer	Magnetron sputtering	N/A	532, 785	4-MBA SAM	[116]
Maskless ICP-RIE SF_6_/O_2_ 10/9 sccm, RF 15 W, 30 mTorr, 200 W ICP, 10 min	(100) p-type, 1−20 Ω · cm 2″	Lace-like, sharp-edged structures: Height: 1 μm, Base width: 100–200 nm, Apex 10–100 nm	Au Pseudo-layer, Approx. 11 nm for side walls, 34–110 nm for apex caps	Magnetron sputtering	1.1 × 10^6^ 4MBA	785	4-MBA, DOX; 10^−9^ M (DOX)	[121]
Cryogenic ICP-RIE SF_6_/O_2_ 30.5/27.5 sccm, 20 mTorr, 4 W platen power, –110 °C	n-type (p-doped), 0.5 mm thick, 100 mm	Cone (pyramidal)-like: Height 495 ± 19, Base diam. 221 ± 24 nm, Apex curvature radius: 26 ± 4 nm	gold 25–50 nm Continuous rough layer	Magnetron sputtering	10^8^	785	4-MBA, living cells C6 rat glioma	[18]
Au pre-coating: - Au coating (e-beam) 5–10 nm, - thermal dewetting. ICP-RIE: etching: SF_6_/O_2_, passivation: C_4_F_8_	(100) 0.2–0.5 Ω·cm, 4″	Truncated cones: Height: 100–500 nm	Prior metallization–Au etched. Ag capping: 5–10 nm Ag backplane (BARNA)	e-beam evaporation, 0.5 Å s^−1^	10^5^–10^6^	633	R6G, nitrate, riboflavin, thiamine, ss-DNA	[67]
Self-masking ICP-RIE SF_6_/O_2_	(100) p-type 4″	Cones (pyramidal)	Intermediate Ti layer 10 nm; Gold layers 100–200 nm; SiO_2_ layer 40 nm; Au NPs 10–60 nm	Magnetron sputtering (Au); ICP CVD (SiO_2_); E-beam evaporation (Au NPs)	N/A	633	R6G, 10^−6^ M	[122]
PS LBL (diam. 500 nm) RIE: CF_4_/Ar: 5/1; 3.33 Pa, RF 60–75 W, 3–40 min	(100) 1 × 1 cm^2^	Au crowned Si pillars	Au 40 nm	Direct current plasma metallization	N/A	533, 633, 785	R6G; MG 10 fM (R6G) Filtered blood plasma (kDa proteins)	[123]

Abbreviations—R6G (rhodamine 6G), 4-MBA (4-mercaptobenzoic acid), SAM (self-assembling monolayer), DOX (doxorubicin), NP (nanoparticle), BARNA (backplane-assisted resonating nanoantenna array), PS LBL (polystyrene Langmuir–Blodgett self-assembly).

## Data Availability

Data are contained within the article.
